# What are we targeting when we support inclusive education for autistic students? A systematic review of 233 empirical studies and call for community partnerships

**DOI:** 10.1177/13623613251352223

**Published:** 2025-08-04

**Authors:** Valentine Perrelet, Aline Veyre, Léa Chawki, Claire Margot, Émilie Cappe

**Affiliations:** 1 Laboratoire de Psychopathologie et Processus de Santé, Université Paris Cité, France; 2 Occupational Therapy Department (HETSL | HES-SO), University of Applied Sciences and Arts Western Switzerland, Switzerland; 3 Faculty of Social Work (HETSL | HES-SO), University of Applied Sciences and Arts Western Switzerland, Switzerland; 4 Institut Universitaire de France, France

**Keywords:** autism spectrum condition, empirical studies, inclusion, inclusive education, systematic review

## Abstract

Interventions to support mainstream schooling for autistic students involve multiple complex considerations. This systematic review, following the Preferred Reporting Items for Systematic Reviews and Meta-Analyses standards, aimed to describe inclusive education interventions, their outcomes, and the tools used to measure the outcomes. A total of 233 experimental studies were included in the review. Subsequent result interpretation was performed using a participatory approach. To this end, a group of consultants comprising lived experience experts participated in two consultation sessions. Most studies were randomized controlled trials, single-case experimental design studies, or other quasi-experimental design studies. The social interactions and social skills of autistic students were the most frequently targeted outcomes. Cognitive-behavioral interventions were the predominant intervention type. A wide, heterogeneous set of standardized instruments was used to measure outcomes, indicating a lack of consensus on how to measure school inclusion and participation. The most used measurement tools assessed core autism symptoms, intelligence quotient, and adaptive behaviors. Approximately half of the studies failed to measure implementation considerations. Participatory interpretation of the results through consultation sessions with lived experience experts enabled the highlighting of interactive processes and considerations involved in inclusive education.

**Lay abstract:**

Multiple complex considerations are involved in supporting mainstream schooling for autistic students. This systematic review aimed to describe inclusive education interventions and outcome measures for autistic students. A total of 233 experimental studies were analyzed. Furthermore, consultation sessions with autistic people, parents, and inclusive education professionals were organized to enable in-depth interpretation of the results using a participatory approach. Cognitive-behavioral interventions were the most common intervention type in the international scientific literature, and social interactions of autistic children were the most frequently targeted outcomes. A lack of consensus on how to measure school inclusion and participation was found. Although some studies considered perspectives on the acceptability of interventions, most of them failed to evaluate implementation aspects. The lived experience experts highlighted tensions between the systematic review findings and the practical realities of inclusive education; this led to discussions about the possible application of the United Nations’ definition of inclusion. Overall, empirical research supporting the inclusion of autistic students aligns more closely with the concept of integration, which requires students to adapt to the school context. The focus of inclusion has been shifting from minimizing the unique traits of students with special needs to adapting the environment for them to take a greater part in school activities.

## Introduction

Inclusive education is now part of the international context of practice guidelines (Farrell, 2000; Frederickson & Cline, 2002; United Nations, 2006). Research has shown that autistic students experience more negative social experiences during schooling compared with other children, including those with other neurodivergent conditions or disabilities (Humphrey & Hebron, 2015; Williams et al., 2019). Furthermore, several studies found that autistic students have lower academic achievement compared with the general population (Ashburner et al., 2010; Keen et al., 2016). Multiple perspectives on the schooling of autistic students, including those of autistic students, parents, and teachers, reveal a complex inclusion process. These perspectives challenge schools to provide effective support systems to include all students, regardless of their disabilities or backgrounds (Bossaert et al., 2013; Hodkinson, 2016). Nevertheless, multiple factors influencing mainstream schooling must be considered. Different levels of influence—from individual characteristics to societal standards, values, and ideologies—hinder school participation for autistic children (Cappe & Boujut, 2016).

Inclusive education may be seen as a process supporting participation, an educational approach supporting teaching practices, or a moral position aimed at equity in society (Florian, 2014). However, articulating the operational meaning of the concept of inclusion is complex, and finding a universally accepted definition that can be applied and evaluated has proven challenging (Artiles et al., 2006). Frameworks designed to capture evidence of inclusive education in action have mostly proved elusive (Florian, 2014). Diverse definitions are necessary to fully understand the complexities of this evolving concept. Several authors have argued that inclusion definitions should be informed by empirical research (Göransson & Nilholm, 2014; Hegarty, 2001; McLeskey et al., 2014).

Describing interventions designed to support an inclusive process enhances the ability to prioritize effective evidence-based support strategies for application in mainstream school settings. Multiple target outcomes can support the general goal of school inclusion. A typology of targeted outcome dimensions derived from experimental research provides scientific insights into what constitutes “inclusive education.” However, internationally available case studies highlight the contextual nature of this process. The prioritization of best practices and the development of general guidelines must be approached with considerable care. Scientific literature often advocates for controlled conditions in building research designs to prove the effectiveness of interventions. Nevertheless, the reality of school-based practice calls for a more integrated research approach that involves multiple partners in decision-making (Grandisson et al., 2014; Parsons et al., 2013).

The interprofessional aspect of monitoring inclusive education involves professionals from a wide range of backgrounds, including health, social work, and education (Bowman et al., 2021). They collaborate with families, who may come from a variety of cultural and socioeconomic backgrounds (Li et al., 2024; Rispoli et al., 2019). Best practice guidelines advocate for interdisciplinary family-centered approaches. Studies addressing interventions within ecological contexts and the challenges of implementing interventions are critical concerns for all partners. In this sense, implementation consideration is as decisive as the availability of evidence-based support measures (Carroll & Peter, 2014).

This study has two main aims. First, through a systematic review, the primary aim is to identify the interventions used to support the inclusion of autistic students in mainstream schools, to provide a comprehensive overview of the outcomes assessed in mainstream schools, and to identify the outcome measures used. The secondary aim of this study is to develop an in-depth interpretation of the results, using a participatory approach. Therefore, the results were discussed with a group of consultants, comprising lived experience experts.

## Systematic review method

This study was conducted following the Preferred Reporting Items for Systematic Reviews and Meta-Analyses (PRISMA) guidelines and checklist to achieve a high standard of reporting (Liberati et al., 2009).

### Information sources

#### Eligibility criteria

Original articles published between 2006 and 2023 were included in the review, with 2006 chosen to coincide with the adoption of the United Nations Convention on the Rights of Persons with Disabilities. Only studies published in English or French (the researchers’ spoken languages) in peer-reviewed scientific journals were included. International studies from diverse geographic locations were considered. Studies were required to include an evaluation of interventions promoting inclusion and/or participation in a school or academic setting. The presence of a control group was optional. All qualitative and quantitative experimental designs were included. Only mainstream compulsory school and general education setting outcomes were considered. Accordingly, the target age range of autistic students was 3 to 18 years old, encompassing a broad range of international mandatory schooling ages. The participants had to be either autistic children according to international diagnosis classifications (*Diagnostic and Statistical Manual of Mental Disorders*, 4th ed., DSM-IV; *Diagnostic and Statistical Manual of Mental Disorders*, 4th ed., text rev., DSM-IV-TR; *Diagnostic and Statistical Manual of Mental Disorders*, 5th ed., DSM-5; International Classification of Diseases, 10th Revision, ICD-10; International Classification of Diseases, 11th Revision, ICD-11) or related partners, such as parents or professionals who support autistic children in inclusive education. No restrictions were imposed based on gender, ethnicity, initial severity of autism spectrum condition, or symptoms related to other neurodevelopmental disorders. Articles describing pharmaceutical treatments, those unrelated to school inclusion, and those focusing on adult education were excluded.

#### Search strategy

A systematic search was performed using multiple large databases in health sciences and psychology: PubMed, PubPsych (including MEDLINE, ERIC, Pascal, PSYNDEX, NARCIS), Cochrane Library, and Embase. The search algorithm was performed in November 2022 through Boolean operators with the following terms: ((mainstream* AND (school OR classroom OR education)) OR (inclus* AND (school OR classroom OR education)) OR (“school participation” OR “classroom participation”) OR (“school engagement” OR “classroom engagement”)) AND (method* OR program* OR curriculum* OR techni* OR therap* OR procedure* OR approach* OR support* OR process* OR intervention* OR framework) AND (autis* OR ASD OR asperger*) NOT (medication* OR drug* OR pharmacotherap* OR pharmaceutic*). The search was completed manually and included Google Scholar, as well as a reference search of retrieved articles. The search was updated through release alerts through November 2023.

#### Selection process

All relevant articles were downloaded. Zotero reference management software was used to import and de-duplicate records. The first and third authors (V.P. and L.C.) independently screened titles and abstracts based on the eligibility criteria. Then, they assessed which articles met the inclusion criteria by reading the full text. Any doubt was resolved through consensus under the supervision of the second and last authors (A.V. and E.C.).

### Data collection

According to the first aim of this study, information describing interventions, major demographic characteristics of the study sample, sample selection and recruitment, targeted outcomes, outcome measures, and dependent and independent variables were extracted from each included study by the first author (V.P.). Data extraction was verified by the third author (L.C.) to mitigate the risk of error.

#### Methodological quality of included studies

Quality was assessed using the Quality Assessment Tool for Observational Cohort and Cross-Sectional Studies (National Institutes of Health, 2016). The instrument comprises 14 items addressing aspects including the research question, study population, sample size justifications, measures, and assessment tools validity. One of three quality levels—good, fair, or low—is assigned to the study according to how these elements affected the methodological quality aspects of the study. The first author (V.P.) assessed the quality of all reports, and the third author (L.C.) assessed 47 (20%) of retrieved reports with 93% inter-rater reliability on the overall grade (Belur et al., 2021).

#### Analysis

A thematic descriptive coding table was developed to meet each objective of the systematic review. The interventions, related reported outcomes, and the measuring tools used in each retrieved paper were recoded multiple times using a thematic analysis method. An initial categorization of 137/233 studies was conducted. Multiple readings of the data made it possible to assess the recurrence of interventions and outcome measures. The categorization was progressive and enabled the grouping in categories based on units of similar meaning and interrelated dimensions. At each of the four iterative categorization stages, the data were grouped into broader categories. All subcategories and terms from the raw data were used to create a definition for each category.^1^ The remaining studies (96/233) were used to check the saturation and relevance of the categorization.

Once the dimensions belonging to the same categories had been grouped by the first author (V.P.), they were reviewed by the second and fourth authors (A.V. and C.M.) to ensure a similar understanding of the different dimensions. The second author reviewed 47 (20%) of the retrieved reports. At this stage, categories were adjusted based on dialogues between the three authors (A.V., C.M., and V.P.). The fourth author (C.M.) then sorted all retrieved papers, reaching a 97% inter-coder reliability with the first author (V.P.). A descriptive statistical analysis was performed to analyze trends in outcome measure assessment and intervention dimensions. The analyses were performed using Rstudio version 24.

## Participatory approach to interpretation of the results

A participatory approach was used to interpret the results through consultation sessions (Jivraj et al., 2014). The group of consultants was recruited using convenience sampling and convened twice. It comprised lived experience experts: four young autistic adults, three parents of autistic children, four leaders and policymakers in the field of special education, professionals in specialized autism services (one head of an educational center for autistic students, one educator, and one special education teacher), and representatives from mainstream schools (one administrative manager for compulsory schools, one mainstream teacher, and two deans from mainstream schools).

To enable participation for all consultants (Bigby et al., 2014), accommodations were provided, including a low-stimulation room for the sessions, use of a respite room, presession meetings, available space description before meetings, directional guidance, and other individual needs accommodations. Two sessions were organized (Jackson et al., 2021; Jivraj et al., 2014; Nind et al., 2021) to allow all lived experience experts to participate in the consultation. The same team of 18 individuals participated in both consultation sessions. To facilitate discussions, lived experience experts were grouped in two different rooms to be able to form groups of 6 to 8 (Hamilton & Finley, 2020). The first consultation session was intended for discussions of interventions and outcomes based on the intermediate results of 137 analyzed studies from the systematic review. The second session further identified results of relevance from the lived experience experts’ perspectives after completing the review of all 233 studies. During the second session, mapping of the results was co-constructed based on the consultants’ initiative. All sessions were audio recorded, and the relevant information was simultaneously collected in a logbook. The main themes on which the experts reached consensus are reported after the results of the descriptive thematic analysis process (Hamilton & Finley, 2020; Jackson et al., 2021; Nind et al., 2021). Given that two of the autistic consultants were unable to attend the sessions, one for personal and the other for organizational reasons, individual meetings based on the same content were conducted with them.

In addition, the experience of the fourth author, who is an autistic research partner, also contributed to this participatory approach. She was particularly involved before and after the consultation sessions, helping to define intervention dimensions, categorize outcome measures, and map results to develop conclusions.

## Systematic review results

**Figure 1.**
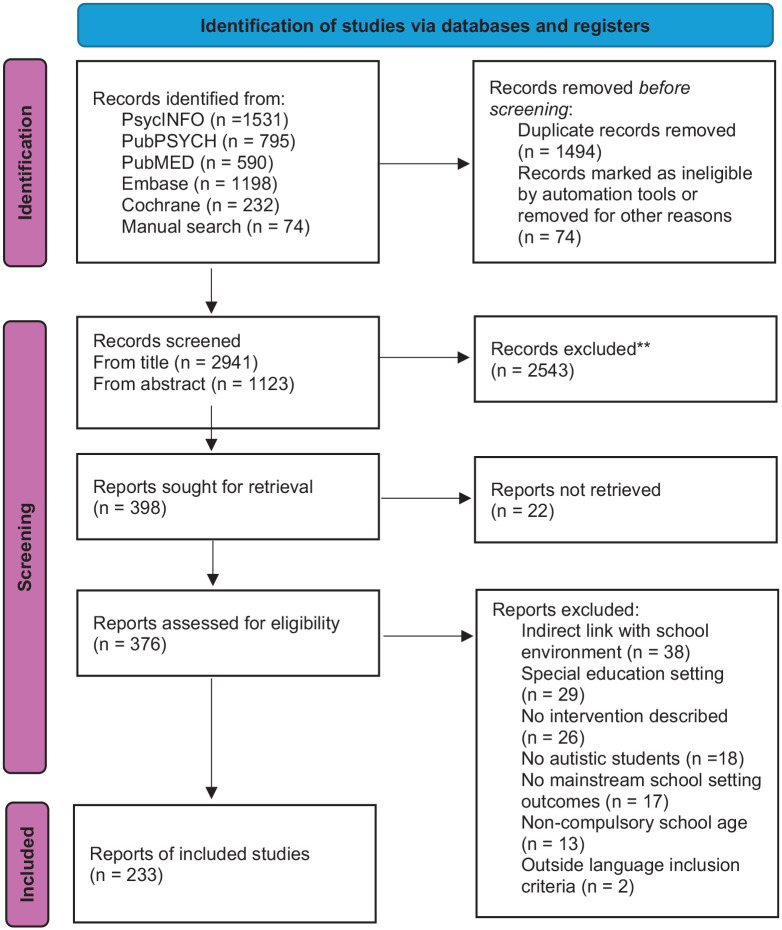
PRISMA flowchart diagram.

The search yielded 2941 records, from which 376 full-text articles were screened. A total of 233 experimental studies were included in the final database. A PRISMA flow diagram is shown in Figure 1 for all selection process reports.^2^ In terms of study methodology, we included 90 single-case experimental design studies (39%), 28 randomized controlled trials (12%), 23 quasi-experimental design studies (10%), 15 case studies (6%), 15 qualitative design studies (6%), 12 exploratory or pilot studies (5%), 12 mixed methods studies (5%), nine comparison controlled trials (4%), six longitudinal studies (3%), four participatory or action research studies (2%), two observational comparative studies (1%), and 17 other isolated experimental research design studies (7%). Table 1 shows the designs of the selected studies.

**Table 1.** Frequent design of retrieved studies.

Most frequent study designs	Frequency
Single case experimental design	90
Randomized controlled trial (RCT)	28
Quasi-experimental design	23
Case study	15
Qualitative design	15
Other experimental research design	13
Exploratory/pilot study	12
Mixed methods	12

Table reports of all methodologies identified in more than 5% of the articles.

**Figure 2.**
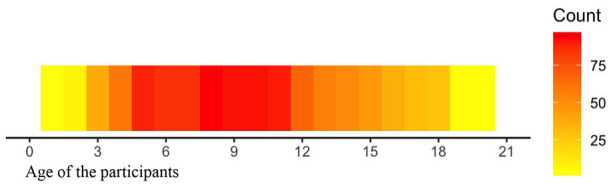
Age range of the autistic participants. Mean = 8 years old (3.17 *SD*); Median = 10 years old.

The studies in our database included 15,535 autistic participants; nevertheless, we acknowledge that the samples may overlap. The mean sample size was 27, with a median of six participants. More than half of the selected studies (*n* = 121; 52%) had a sample size of less than 10 autistic participants. Female participants represented an average of 18.2% of the sample size. One study identified three individuals with gender-diverse profiles. The age range of the autistic participants was between 1 and 20 years old, extending beyond the inclusion criteria to avoid losing part of the sample. Most of the included studies targeted an age range between 5 and 11 years old, with 8 years old being the most frequent target participant age (*n* = 97). Figure 2 shows the age ranges of participants.^3^ Of the 233 studies, 94 included other target populations, such as peers (*n* = 44), mainstream teachers (*n* = 31), parents (*n* = 9), and other school personnel (*n* = 28), such as educators, special education teachers, principals, co-teachers, and other practitioners.^4^ Coexisting conditions of autistic participants were recorded in 24 studies, including intellectual development disorders (*n* = 4), blindness (*n* = 4), attention-deficit hyperactivity disorder (*n* = 2), specific learning disorder with impairment in reading (*n* = 1), and other or unspecified developmental disorders (*n* = 9). Five studies included only high-functioning autism.^5^

The locations of the research spanned 59 countries. The studies were primarily conducted in the United States (*n* = 112), the United Kingdom (*n* = 24), and Australia (*n* = 21).^6^ Publication numbers since 2006 were generally increasing, with a maximum of 30 articles published in 2019 and a minimum of four in 2008 and 2011. We selected a total of 36 articles from 2006 to 2011, 86 from 2012 to 2017, and 111 from 2018 to 2023.^7^ According to the method described using the Quality Assessment Tool for Observational Cohort and Cross-Sectional Studies (National Institutes of Health, 2016), 30 (13%) of the studies were assessed as good quality, 96 (41%) were scored as fair, and 107 (46%) were deemed to have low reporting quality.

### Interventions targeting school participation and inclusion

Most of the described interventions were implemented directly in mainstream classroom settings (*n* = 124), in an adjacent room in the school building or an empty classroom (*n* = 44), or in another school facility, such as a gymnasium, school playground, or cafeteria (*n* = 22). Although the objectives were focused on school inclusion, some of the interventions occurred in other locations, such as childcare settings (*n* = 3), home (*n* = 3), or another out-of-school environment (*n* = 14).^8^ Interventions focusing on professional development occurred at school or in different locations, including online (*n* = 7). All interventions aimed at improving school inclusion or participation were analyzed, regardless of their location.

**Figure 3.**
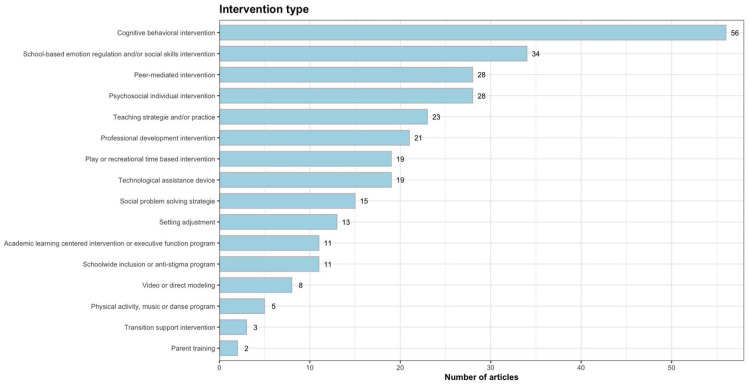
Type of interventions and their distribution.

The types of interventions aimed at school participation and inclusion in the studies were classified based on the main components of the procedures described (see Figure 3).^9^ Cognitive-behavioral interventions were the most prevalent (*n* = 56), followed by school-based social skills and emotion regulation interventions (*n* = 34); peer-mediated interventions (*n* = 28); psychosocial individual interventions (*n* = 28); teaching strategies and practices (*n* = 23); professional development (*n* = 21); play or recreational time-based interventions (*n* = 19); technological assistance devices (*n* = 19); social problem-solving strategies, such as social scripts (*n* = 15); setting adjustments (*n* = 13); academic learning-centered interventions or executive function programs (*n* = 11); schoolwide inclusion or anti-stigma programs (*n* = 11); videos or direct modeling (*n* = 8); physical activity, music, or dance programs (*n* = 5); transition support intervention (*n* = 3); and parental training for school readiness or school support (*n* = 2). A total of 60 experimental studies included multiple types of interventions, and 11 were found to have more than two types of component interventions.

### Outcome measures in the context of school inclusion

**Figure 4.**
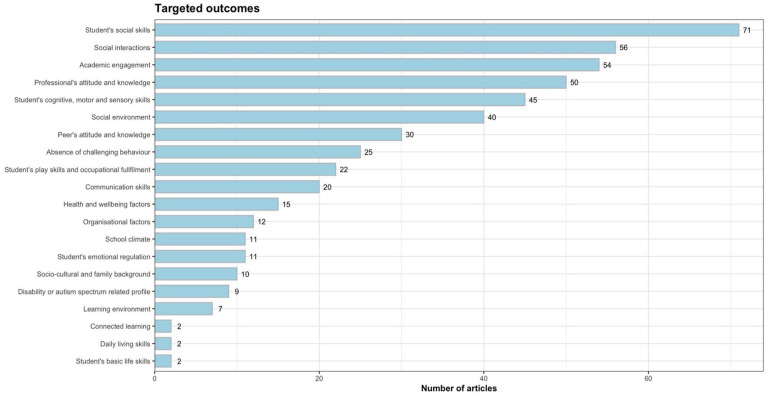
Dimensions of outcome measures.

Outcome measures covered many areas and were classified according to the dimensions of school participation and inclusion. Of the 233 studies, 195 used more than one dependent variable, and 120 targeted more than two. Dimension classifications are listed in Figure 4.^10^ The outcome dimensions were primarily directed toward student social skills (*n* = 71) or social interactions (*n* = 56). In addition, academic engagement (*n* = 54) and professionals’ attitudes and knowledge (*n* = 50) were common outcome types. Outcomes targeting students’ cognitive, motor, and sensory skills (*n* = 45) and social environment factors (*n* = 40), such as social validity or feasibility of implementation, were present. A few studies addressed peer attitudes and knowledge (*n* = 30), the reduction of challenging behavior (*n* = 25), students’ play skills and occupational fulfillment (*n* = 22), and communication skills (*n* = 20). The least represented types of outcomes were health and well-being (*n* = 15), organizational factors (*n* = 12), school climate (*n* = 11), students’ emotional regulation (*n* = 11), sociocultural and family background (*n* = 10), and disability or autism-related profiles (*n* = 9).

#### Type and occurrence of assessment tools

A total of 145 different standardized outcome measures were found. Most were employed in a single study (*n* = 115), and 20 others were found in less than five different studies. The Autism Diagnostic Observation Schedule (ADOS; Lord et al., 2000) was used in 15 studies, most often as a screening tool. The Wechsler Preschool and Primary Scale of Intelligence was found in different versions: WPPSI-R, WIPPSI-III, WIPPSI-IV, WASI, and the Hong Kong or Chinese version (Li et al., 2011; Wechsler, 1989, 2002, 2012). The Vineland Adaptive Behavior Scales, VABS or VABS-II (Sparrow et al., 1984, 2005), were used in 12 studies. The Childhood Autism Rating Scale, CARS, CARS-2, or the Chinese version (Lu et al., 2004; Schopler et al., 1986, 2010), was used in 11 studies. The Social Skills Improvement System Rating Scales (SSIS-RS; Gresham & Elliot, 2008) were used in eight studies. The Stanford–Binet Intelligence Scales, SBS-III, SBS-V, or the Chinese version (Roid, 2003; Terman & Merrill, 1973; Yu, 2004), were used in six studies. The Social Responsiveness Scale (SRS-2; Constantino & Gruber, 2012), Autism Diagnostic Interview–Revised (ADI-R; Rutter et al., 2003), Differential Ability Scales (DAS or DAS-II; Elliott, 1990, 2007), and Intervention Rating Profiles (IRP or IRP-REI; Rakap, 2017; Witt & Martens, 1983) were found in five different studies each. The outcome measures are presented according to the second objective of the study.^11^ The evaluation measures targeting aspects of school participation or inclusion were extremely heterogeneous, with none retrieved from more than five studies.

## Participatory interpretation of the results

Consultation with the lived experience experts enabled meaningful interpretation of the results. The outcomes and interventions were interpreted from their perspectives. Mapping was performed in the group of consultants to illustrate interactions and considerations of the different dimensions previously discussed. A collaborative activity enabled all lived experience experts to diagram their thoughts and highlight interactive processes involved in inclusive education.

The consultants identified further considerations, possible interactions, and overlap between the categorized dimensions. They mentioned that scientific research frequently overlooks the political and sociocultural aspects that are closely linked to school inclusion. Examples given by consultants were the conventional and legal contexts, individual perspectives and opinions about inclusion, and budget allocation for specific inclusive school-based interventions. Although aspects of feasibility and implementation were considered in some studies (*n* = 77 and *n* = 20 with incomplete measures or implicit mentions), fluctuating economic and political systems extremely complicate the implementation of evidence-based interventions. The reported interventions primarily focused on the child’s skills, with some emphasizing environmental adaptations (*n* = 117). A meta-result of this process concerns the heterogeneity of priorities in relation to the outcomes targeted by the interventions in the school inclusion context. Education, health, and pedagogy professionals stress the importance of early intervention, whereas parents prioritize their children’s mental health and well-being. Autistic individuals seem more sensitive to professionals’ attitudes and knowledge.

**Figure 5.**
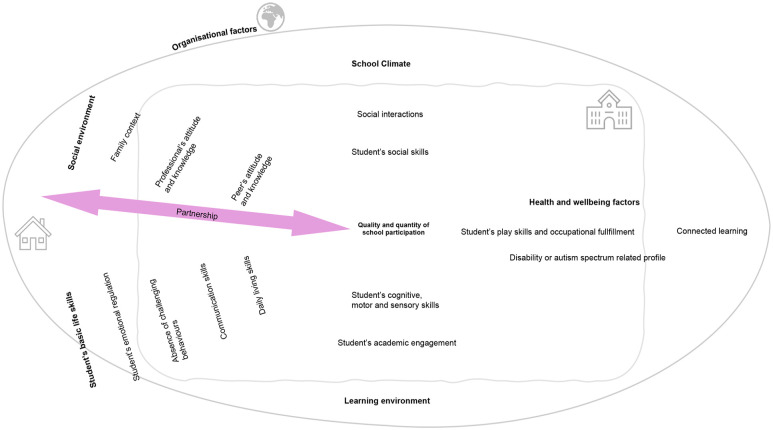
Mapping of school inclusion dimensions.

Figure 5 describes the mapping intervention dimensions based on the input of the group of consultants. The diagram shows the prioritizing of dimensions as defined by organizational context. The reflection about interaction complexity is represented at every level, from environmental to individual factors. In the center, school inclusion is defined by the quantity and quality of school participation. At the top and bottom are two funnels depicting the interconnectivity between school connectedness and the social skills of autistic students, on the one hand, and the learning environment and students’ cognitive motor and sensory skills, on the other hand. These systems interact through bidirectional links between students and their environment. Similarly, the square shows two dimensions referring to the school environment. The oval refers to the children’s daily living environments (with a house representing the family context). Organizational factors refer to general policies and practices in the environment. The horizontal axis shows cross-cutting competencies related to the students’ daily lives, and collaboration with all partners at the school and in the family environment is shown. According to the consultants’ input, the timeline represented by the arrow shows the direction in which inclusive schools should focus.

This attempt at prioritizing and mapping core dimensions from an empirical perspective reveals discrepancies between the frequency of interventions reported and priorities identified by key partners during consultation sessions.

## Discussion

This study aimed to identify interventions and outcomes to support inclusive education for autistic students, as well as discuss inclusive education with autistic and school community partners using a participatory approach. A total of 233 experimental studies, using a variety of methods, were reviewed. The most frequent intervention types were cognitive-behavioral interventions. Intervention and outcome analysis allowed the consultants to map the core dimensions of the school inclusion process. The social interactions and social skills of autistic children were the most frequently targeted outcomes.

Most studies were single-case experimental design studies, randomized controlled trials, or other quasi-experimental design studies. To report the quality of interventions in community settings, studies need to be replicated using the same outcome measure. Maggin et al. (2014) emphasized the need for single-case studies to be replicated with similar methodological criteria. To measure outcomes, a wide, heterogeneous set of standardized instruments was retrieved from the selected studies. A large majority of reported measures were used in less than three studies, representing a lack of consensus in measuring school inclusion and participation constructs.

The most frequently used measurement tools were those assessing the core symptoms of autism, intelligence quotient, and adaptive behaviors. Although multiple perspectives on the implementation aspects of interventions were found in some studies, most did not consider these aspects. Similar to other autism spectrum–related review studies (Benevides et al., 2020), consultation sessions with autistic partners, parents, and inclusive education professionals helped contextualize the results, highlighting key challenges and resources. Including lived experience experts in this research was essential to enable the scientific communities to understand the priorities and perceptions of autistic people, their families, and inclusive education professionals, including their views on the existing or available approaches. The review of the findings by the group of consultants identified tensions between the systematic review findings and the practical realities of inclusive education.

Considering the progression of inclusion movements (Kivirauma et al., 2006), the emphasis is on fostering a democratic society that offers a comprehensive school system for all. The evolution of the concept of integration toward that of inclusion primarily emphasizes every child’s right to participate in a common education. The focus has shifted from minimizing the unique traits of children with special needs to adapting the environment to enhance their ability to act and participate (Allodi, 2002; Thomas & Loxley, 2001). Our findings indicate that empirical research supporting the inclusion of autistic students aligns more closely with the concept of integration, which requires children to adapt to the school context by acquiring new skills and modifying their behavior. In fact, assessments primarily focused on core autism symptoms, intellectual quotient, and adaptive behaviors. The consulted lived experience experts confirmed that situations experienced in the school context are closely related, from the perspectives of professionals, autistic individuals and their families. The scarcity of interventions focused on physical, social, and sensory environment adaptation reveals a gap between the definition of inclusion according to the United Nations and current research practices, as well as everyday school practices. Moreover, the influence of the sensory and physical environment on students’ school life, learning, and behavior has been demonstrated by several authors (Al Qutub et al., 2024; Jones et al., 2020). Creating good sensory conditions enables the inclusion of autistic students and benefits everyone (Martin et al., 2019). Our findings align with those of Carrington et al. (2020), suggesting that the development of support based on universal design concepts is not yet evident in inclusive education when supporting autistic students. Indeed, few analyzed studies mentioned broader population coverage.

Prioritizing the dimensions of the interventions and mapping the processes of interaction between the various factors derived from the outcome measures have enabled the development of a model based on scientific literature that incorporates the perspectives of those concerned. However, the diverse profiles of the inclusive education partners represented in the consultation sessions have led to differing opinions about the analysis and interpretation of some results. Following the example of Bigby and Frawley (2010), the team members adopted a supportive stance, recognizing the resources of all its members and allowing all points of view to be challenged, regardless of the implicit and explicit hierarchies in force. A consensus was reached after several back-and-forth discussions.

As the social interactions and social skills of autistic students were the main target outcome in the reviewed scientific papers, the group of consultants included perspectives centered on stigma breakdown from the political level to school cultures. Previous contributors, examining closely related aspects such as social relations at school (Dean & Chang, 2021) or academic success (Keen et al., 2016; Leifler et al., 2021) for autistic students, reached similar conclusions regarding the need to focus on environmental factors. Parents in our consultation sessions noted that ongoing efforts are needed to promote effective and meaningful collaboration with families in the context of school participation and orientation. In some scenarios, parents must extend their role to support their child in the school context, which creates significant burdens (Parish et al., 2015) and increases the risk of parental stress, depression, and burnout (Bourke-Taylor et al., 2010). These effects, in turn, have secondary effects on the child’s situation (Herring et al., 2006). Few studies have considered parental perspectives, whether in the intervention phase or in effectiveness and implementation evaluations. Although experts and guidelines agree on the importance of family-centered practice, the recognition of parental expertise in educational interventions related to autism spectrum condition requires further investigation (Hodgetts et al., 2015; Yon, 2020). Consistent with other studies (Boer et al., 2005; Roberts & Simpson, 2016), autistic consultants also emphasized the importance of considering their schooling experiences.

The scarcity of schoolwide programs and other interventions based on environmental adaptation could be due to the complexity of measurement variables in a community context, such as a mainstream school. Previous review studies examining interventions in the field of autism spectrum have also found great heterogeneity in outcome measurements (Provenzani et al., 2020). We found that most of the measurement tools were unique, with only a few standardized tools being consistent across countries and contexts. These tools primarily focused on student skills and individual characteristics.

The methodological challenge of implementing interventions in ecological environments may explain this phenomenon. Establishing conditions to effectively evaluate community-based interventions has proved to be challenging (Finkenflugel et al., 2005). As described by Grandisson et al. (2014), a community-based evaluation process offers documented advantages, as well as specific time and resource constraints for researchers considering this approach. Their findings indicate that, despite several existing resources, no consensual framework has been established and that the combination of quantitative and qualitative methods has significant potential to capture the effectiveness of community interventions more accurately. In addition, the evaluation process should be conducted in close collaboration with all partners of the autistic and school communities (Grandisson et al., 2014; Parsons et al., 2013).

Developing implementation strategies, in conjunction with the expansion of participatory methods, facilitates the consideration of perspectives from all involved partners. Supporting school personnel in adopting evidence-based inclusive practices tailored to their needs helps mitigate the risk of burnout (Rajotte et al., 2023). Although incorporating the opinions of all partners adds complexity, Kazdin (2011) emphasized that it should be the main concern if efficiency is to be increased. Implementation should be assessed through several data collection methods, dimensions, and informants.

Research and educational practice in the field of inclusive schooling should therefore be centered on the recognition and empowerment of neurodiversity. A clear priority for further research is the adaptation of settings before expecting students to meet preestablished criteria, as highlighted by international guidelines (United Nations, 2006), scientific literature (Roche et al., 2021), and the results of this study. This approach offers operational strategies that align with all levels of the conceptual definition of inclusion inspired by the United Nations (2006). It supports a process that promotes participation, including educational approaches aimed at fostering a moral position based on equity (Florian, 2014). Based on the consultants’ review of findings, key components toward building more inclusive mainstream schools may involve the following: strengthening environmental adaptation; promoting evidence-based practice through professional development; implementing relevant monitoring of inclusion and school participation; enhancing collaboration between families, schools, and external services; and further developing community-based research involving all partners’ perspectives.

As advised by fellow academics (Göransson & Nilholm, 2014; McLeskey et al., 2014), this systematic review has contributed to building and mapping a definition of inclusive education for autistic students based on empirical research. The retrieved experimental studies and the participatory consultation sessions have complementarily contributed to elucidating the dimensions involved in school inclusion and their prioritization based on current scientific evidence and practical realities.

### Limitations

The current synthesis is, to our knowledge, the most comprehensive systematic review of interventions and outcome measures applied to inclusive education for autistic students. However, limitations should be discussed. This review did not analyze intervention effectiveness or single tools by comparing their availability or psychometric properties. Therefore, the studies were not weighted according to their quality ratings.

Previous studies and reviews have already extensively explored interventions targeting specific domains in the school context, from the perspectives of social participation (Bossaert et al., 2013; Dean & Chang, 2021; Watkins et al., 2019), academic achievement (Haas et al., 2020; Keen et al., 2016; Ledbetter-Cho et al., 2023), and behavioral interventions (Pellecchia et al., 2016). Furthermore, issues regarding the assessment and reporting of findings, along with reported biases in trial outcomes, have been described by Sandbank et al. (2024). Therefore, this review does not enable conclusions regarding optimal interventions or tools but rather offers an overview of their utilization patterns and descriptions of their core components.

A further potential limitation of our analysis relates to the characteristics of the included studies: most studies did not adequately report important data, especially sample characteristics such as other neurodevelopmental conditions, socioeconomic status, ethnicity, gender, or the educational attainment levels of professionals or parents. Therefore, analyses considering socioeconomic status, ethnicity, and educational levels were not described. The hypotheses derived from the analyses of gender and concurrent neurodevelopmental conditions should be considered cautiously. Autism spectrum conditions include a range of heterogeneous characteristics; therefore, the effectiveness of an intervention may be influenced by identifying precisely for which subpopulation it is designed. The quality of the retrieved and analyzed studies was mostly poor, and this review included interventions with significant limitations, such as reporting biases and lack of congruency. An in-depth analysis of the data according to certain parameters, such as age, location of intervention, or geopolitical context, was not conducted due to data reporting bias, the heterogeneity of represented locations of study, and the wide age range of participants. In addition, only nonmedical interventions were considered. Nevertheless, medical interventions directly linked to the context of inclusive education are scarce and not featured in international guidelines (Guldberg et al., 2011; Provenzani et al., 2020). Given the vast number of reports retrieved for this in-depth conceptual systematic review, the process was conducted over multiple years, with the last date of search in 2023. Considering the rapid growth of research on related topics, we acknowledge the possibility that some literature might be absent from this study due to factors such as not being indexed at the time of search, not being captured by the specific search criteria used, or being in press; thus, future analyses should be conducted.

## Conclusion

The results of this systematic review, complemented by a participatory consultation, have contributed to redefining inclusion in empirical research based on studies focused on inclusive education for autistic students. Our findings suggest that due to the numerous heterogeneous factors involved in studies targeting inclusive education, no single measurement tool captures all relevant variables. This is particularly apparent when considering the need to adjust assessment measures for different settings. Significant contextual variations and challenges associated with evaluating community-based interventions highlight the complexity of experimental research in this field. Moreover, ongoing politico-cultural evolutions and the development of ideological concepts linked to inclusive education create continually changing conditions. The selection of outcome measures remains very broad, which limits synthesis and generalization and reveals considerable difficulties in choosing specific target domains. This increases the difficulty of linking theory, applied research, and interventional practices. Therefore, using various instruments simultaneously and combining data collection methods could be beneficial, given the lack of consensus regarding reliable standardized outcome measures. Furthermore, underexplored but crucial areas of inclusive education, such as school–health–family partnerships, require further consideration.

## Supplemental Material

sj-docx-1-aut-10.1177_13623613251352223 – Supplemental material for What are we targeting when we support inclusive education for autistic students? A systematic review of 233 empirical studies and call for community partnerships Supplemental material, sj-docx-1-aut-10.1177_13623613251352223 for What are we targeting when we support inclusive education for autistic students? A systematic review of 233 empirical studies and call for community partnerships by Valentine Perrelet, Aline Veyre, Léa Chawki, Claire Margot and émilie Cappe in Autism

## Notes

1. The categories of intervention dimensions and targeted outcomes are defined in the Supplemental material.

2. A complete list of included studies is available in the Supplemental material.

3. For further details on the age range of participants, see the Supplemental material.

4. Figures are available in the Supplemental material.

5. Referring to high-functioning autism is no longer recommended (Alvares et al., 2020); the term has been primarily used in older studies.

6. A figure presenting the geographic origins of the studies is available in the Supplemental material.

7. A figure showing the publication years of the selected articles is available in the Supplemental material.

8. Details about intervention locations are available in the Supplemental material.

9. The categories of targeted outcomes are defined in the Supplemental material.

10. The core intervention dimensions are defined in the Supplemental material.

11. The outcome measures reported in five or more studies are listed in the Supplemental material.
