# Supplementary material for: What are we targeting when we support inclusive education for autistic students? A systematic review of 233 empirical studies and call for community partnerships
Source: Autism. 2025 Aug 4;29(12):2927–40. doi: 10.1177/13623613251352223 (PMC12618732; doi:10.1177/13623613251352223)
Supplement: sj-docx-1-aut-10.1177_13623613251352223 – Supplemental material for What are we targeting when we support inclusive education for autistic students? A systematic review of 233 empirical studies and call for community partnerships [file sj-docx-1-aut-10.1177_13623613251352223.docx]

# Supplementary material

Figure 1: Age range of participants

Figure 2: Other target populations

Figure 3: Geographic origins of selected studies

Figure 4: Year of publication of selected articles

Figure 5: Location of interventions

Figure 6: School inclusion dimensions

Table 2: Most frequent standardized measuring tools

Complete list of included studies

## Figure 1: Age range of participants

**
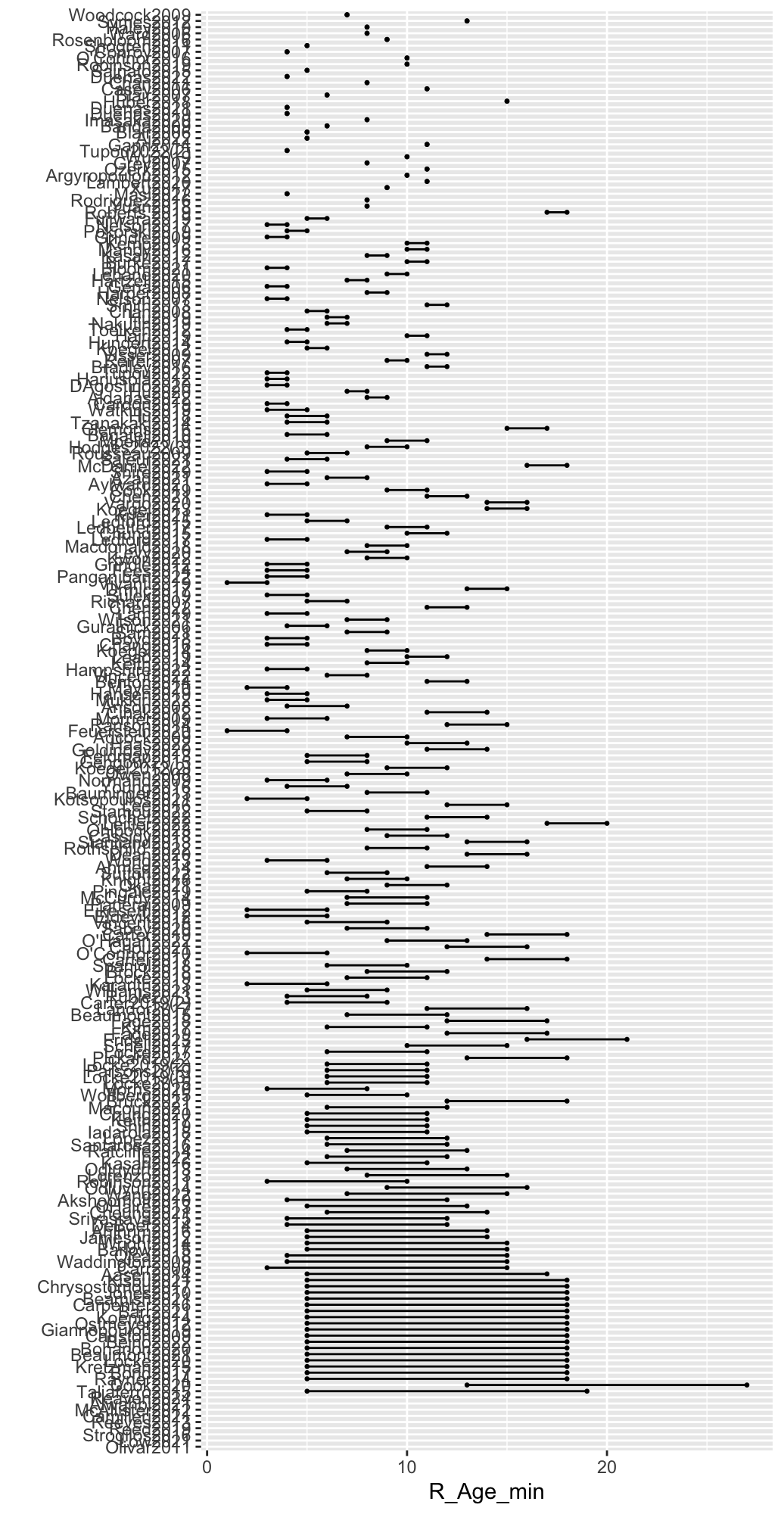
**

Retrieved reports

Age range

## Figure 2: Other target populations

Number of articles targeting the population

## Figure 3: Geographic origins of selected studies


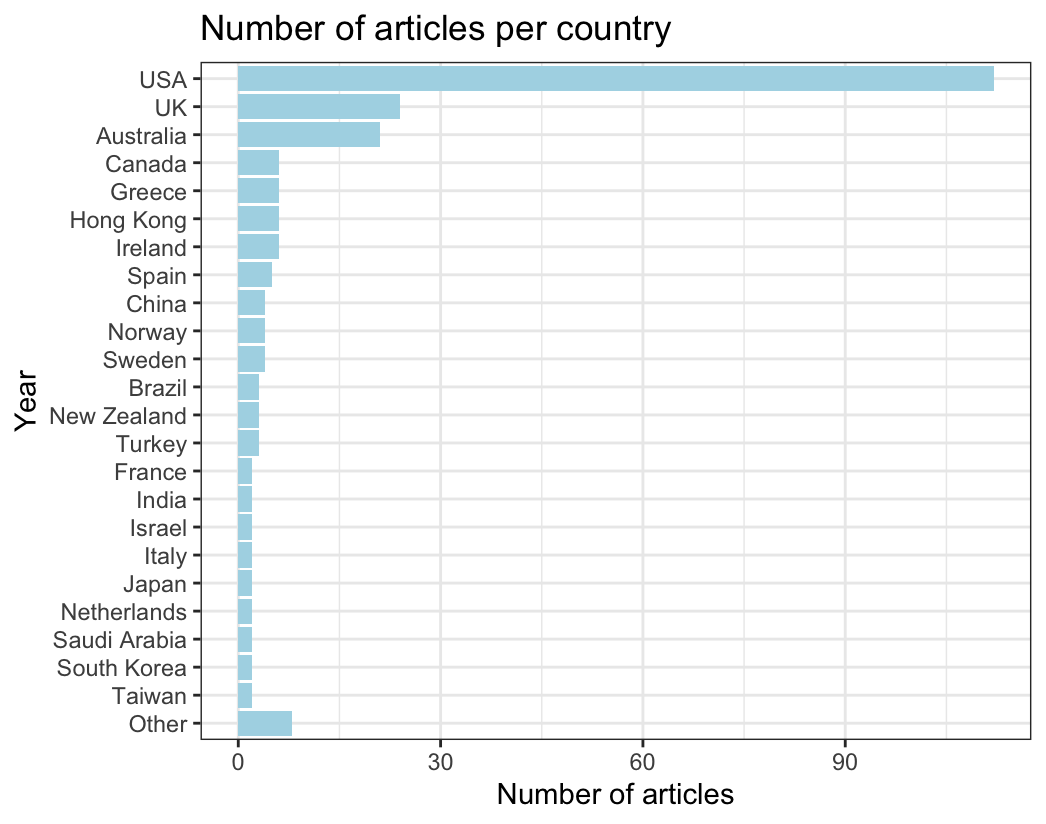


## Figure 4: Year of publication of selected articles

Number of articles

Year of publication

## Figure 5: Location of interventions

Number of articles

## Figure 6: School inclusion dimensions

## Table 2: Most frequent standardized measuring tools

| Measuring tool | Frequency |
| --- | --- |
| Wechsler Preschool and Primary Scale of Intelligence (WPPSI) | 15 |
| Autism Diagnostic Observation Schedule (ADOS) | 15 |
| Vineland Adaptive Behavior Scales (VABS) | 12 |
| Childhood Autism Rating Scale (CARS) | 11 |
| Social Skills Improvement System Rating Scales (SSIS- RS) | 8 |
| Stanford–Binet Intelligence Scales (SBS) | 6 |
| Intervention Rating Profiles (IRP) | 5 |
| Differential Ability Scales (DAS) | 5 |
| Social Responsiveness Scale (SRS) | 5 |
| Autism Diagnostic Interview-Revised (ADI-R) | 5 |

## Complete list of included studies

Aasen, G., & Nærland, T. (2014). Enhancing activity by means of tactile symbols : A study of a heterogeneous group of pupils with congenital blindness, intellectual disability and autism spectrum disorder. *Journal of Intellectual Disabilities: JOID*, *18*(1), 61‑75. <https://doi.org/10.1177/1744629514522142>

Adcock, J., & Cuvo, A. J. (2009). Enhancing learning for children with autism spectrum disorders in regular education by instructional modifications. *Research in Autism Spectrum Disorders*, *3*(2), 319‑328. <https://doi.org/10.1016/j.rasd.2008.07.004>

Ahlgrim-Delzell, L., Browder, D. M., Wood, L., Stanger, C., Preston, A. I., & Kemp-Inman, A. (2016). Systematic instruction of phonics skills using an iPad for students with developmental disabilities who are AAC users. *The Journal of Special Education*, *50*(2), 86‑97. <https://doi.org/10.1177/0022466915622140>

Ahmed-Husain, S., & Dunsmuir, S. (2014). An evaluation of the effectiveness of Comic Strip Conversations in promoting the inclusion of young people with autism spectrum disorder in secondary schools. *International Journal of Developmental Disabilities*, *60*(2), 89‑108. <https://doi.org/10.1179/2047387713Y.0000000025>

Ai, J., Zhao, M., Behrens, S., & Horn, E. M. (2022). Professional development improves teachers’ embedded instruction and children’s outcomes in a chinese inclusive preschool. *Journal of Behavioral Education*. <https://doi.org/10.1007/s10864-022-09490-5>

Akshoomoff, N., Stahmer, A. C., Corsello, C., & Mahrer, N. E. (2010). What Happens Next? Follow-Up From the Children’s Toddler School Program. *Journal of Positive Behavior Interventions*, *12*(4), 245‑253. <https://doi.org/10.1177/1098300709343724>

Aldabas, R. (2022). Effects of peer network intervention through peer-led play on Basic Social Communication Skills of children with Autism Spectrum Disorder in Inclusive Classroom. *Journal of Developmental and Physical Disabilities*, *34*(6), 1121‑1148. <https://doi.org/10.1007/s10882-022-09840-1>

Alwahbi, A., & Hua, Y. (2021). Using Contingency Contracting to Promote Social Interactions Among Students With ASD and Their Peers. *Behavior modification*, *45*(5), 671‑694. <https://doi.org/10.1177/0145445520901674>

Anson, H. M., Todd, J. T., & Cassaretto, K. J. (2008). Replacing overt verbal and gestural prompts with unobtrusive covert tactile prompting for students with autism. *Behavior Research Methods*, *40*(4), 1106‑1110. <https://doi.org/10.3758/BRM.40.4.1106>

Argyropoulou, Z., & Papoudi, D. (2012). The training of a child with autism in a Greek preschool inclusive class through intensive interaction : A case study. *European Journal of Special Needs*, *27*(1), 99‑114. <https://doi.org/10.1080/08856257.2011.640489>

Aylward, E., & Neilsen-Hewett, C. (2021). Application of an evidence-based early intervention model for children with ASD in mainstream early childhood education and care settings via a targeted professional development program. *Australasian Journal of Special and Inclusive Education*, *45*(2), 135‑149. <https://doi.org/10.1017/jsi.2021.11>

Azad, G. F., Minton, K. E., Mandell, D. S., & Landa, R. J. (2021). Partners in School : An Implementation Strategy to Promote Alignment of Evidence-Based Practices Across Home and School for Children with Autism Spectrum Disorder. *Administration and policy in mental health*, *48*(2), 266‑278. <https://doi.org/10.1007/s10488-020-01064-9>

Bagatell, N., Mirigliani, G., Patterson, C., Reyes, Y., & Test, L. (2010). Effectiveness of Therapy Ball Chairs on Classroom Participation in Children With Autism Spectrum Disorders. *The American Journal of Occupational Therapy*, *64*(6), 895‑903. <https://doi.org/10.5014/ajot.2010.09149>

Banda, D. R., Hart, S. L., & Liu-Gitz, L. (2010). Impact of training peers and children with autism on social skills during center time activities in inclusive classrooms. *Research in Autism Spectrum Disorders*, *4*(4), 619‑625. <https://doi.org/10.1016/j.rasd.2009.12.005>

Barlow, A., Humphrey, N., Lendrum, A., Wigelsworth, M., & Squires, G. (2015). Evaluation of the implementation and impact of an integrated prevention model on the academic progress of students with disabilities. *Research in Developmental Disabilities*, *36*, 505‑525. <https://doi.org/10.1016/j.ridd.2014.10.029>

Barr, A., Coates, E., Kingsley, E., De La Cuesta, G. G., Biggs, K., Le Couteur, A., & Wright, B. (2022). A mixed methods evaluation of the acceptability of therapy using LEGO ® bricks ( LEGO ® based therapy) in mainstream primary and secondary education. *Autism Research*, *15*(7), 1237‑1248. <https://doi.org/10.1002/aur.2725>

Bauminger-Zviely, N., Eden, S., Zancanaro, M., Weiss, P. L., & Gal, E. (2013). Increasing social engagement in children with high-functioning autism spectrum disorder using collaborative technologies in the school environment. *Autism*, *17*(3), 317‑339. <https://doi.org/10.1177/1362361312472989>

Beamish, W., Taylor, A., Macdonald, L., Hay, S., Tucker, M., & Paynter, J. (2021). Field testing an Australian model of practice for teaching young school-age students on the autism spectrum. *Research in developmental disabilities*, *113*, 103942. <https://doi.org/10.1016/j.ridd.2021.103942>

Beaumont, D., Blakey, T., Stuart, N., & Woodward, J. (2021). Increasing Engagement for Young Children With Autism Spectrum Disorder Using Way to Play : A Preliminary Investigation of the Adult Training Program. *Australasian Journal of Special and Inclusive Education*, *45*(2), 178‑190. <https://doi.org/10.1017/jsi.2021.14>

Beaumont, R., Rotolone, C., & Sofronoff, K. (2015). The secret agent society social skills program for children with high‐functioning autism spectrum disorders : a comparison of two school variants. *Psychology in the Schools*, *52*(4), 390‑402. <https://doi.org/10.1002/pits.21831>

Bejnö, H., Bölte, S., Linder, N., Långh, U., Odom, S. L., & Roll-Pettersson, L. (2022). From Someone Who May Cause Trouble to Someone You Can Play With : Stakeholders’ Perspectives on Preschool Program Quality for Autistic Children. *Journal of Autism and Developmental Disorders*, *52*(9), 3890‑3908. <https://doi.org/10.1007/s10803-021-05268-2>

Benton, L., & Johnson, H. (2014). Structured approaches to participatory design for children : Can targeting the needs of children with autism provide benefits for a broader child population? *Instructional Science*, *42*(1), 47‑65. <https://doi.org/10.1007/s11251-013-9297-y>

Blair, K.-S., Liaupsin, C., Umbreit, J. & Kweon, G. (2006). Function-Based Intervention to Support the Inclusive Placements of Young Children in Korea. *Education and Training in Developmental Disabilities*, 41(1), 48-57.

Blair, K.-S. C., Umbreit, J., Dunlap, G., & Jung, G. (2007). Promoting inclusion and peer participation through assessment-based intervention. *Topics in Early Childhood Special Education*, *27*(3), 134‑147. <https://doi.org/10.1177/02711214070270030401>

Bloom, L. P. (2021). Professional development for enhancing autism spectrum disorder awareness in preschool professionals. *Journal of Autism and Developmental Disorders*, *51*(3), 950‑960. <https://doi.org/10.1007/s10803-020-04562-9>

Bohanon, H. S., & Wu, M.-J. (2020). A comparison of sampling approaches for monitoring schoolwide inclusion program fidelity. *International Journal of Developmental Disabilities*, *66*(3), 204‑213. <https://doi.org/10.1080/20473869.2018.1546793>

Bond, C., Hebron, J., & Oldfield, J. (2017). Professional learning among specialist staff in resourced mainstream schools for pupils with ASD and SLI. *Educational Psychology in Practice*, *33*(4), 341‑355. <https://doi.org/10.1080/02667363.2017.1324406>

Boyd, B. A., Watson, L. R., Reszka, S. S., Sideris, J., Alessandri, M., Baranek, G. T., Crais, E. R., Donaldson, A., Gutierrez, A., Johnson, L., & Belardi, K. (2018). Efficacy of the ASAP Intervention for Preschoolers with ASD : A Cluster Randomized Controlled Trial. *Journal of Autism and Developmental Disorders*, *48*(9), 3144‑3162. <https://doi.org/10.1007/s10803-018-3584-z>

Bradley, R. (2016). ‘Why single me out?’ Peer mentoring, autism and inclusion in mainstream secondary schools : ‘Why single me out?’ Peer mentoring, autism and inclusion in mainstream secondary schools. *British Journal of Special Education*, *43*(3), 272‑288. <https://doi.org/10.1111/1467-8578.12136>

Brock, M. E., Dueker, S. A., & Barczak, M. A. (2018). Brief Report : Improving Social Outcomes for Students with Autism at Recess Through Peer-Mediated Pivotal Response Training. *Journal of Autism and Developmental Disorders*, *48*(6), 2224‑2230. <https://doi.org/10.1007/s10803-017-3435-3>

Bruck, S., Robinson, A., & Gallagher, E. (2021). A Model of Practice for Improving Autism Knowledge in Teachers of Mainstream Students on the Autism Spectrum in Australia. *Australasian Journal of Special and Inclusive Education*, *45*(2), 221‑236. <https://doi.org/10.1017/jsi.2021.7>

Burke, M.-K., Prendeville, P., & Veale, A. (2017). An evaluation of the “FRIENDS for Life” programme among children presenting with autism spectrum disorder. *Educational Psychology in Practice*, *33*(4), 435‑449. <https://doi.org/10.1080/02667363.2017.1367648>

Camilleri, L. J., Maras, K., & Brosnan, M. (2021). Mothers’ and practitioners’ insights on the use of digitally-mediated social stories with children on the autism spectrum : A convergent mixed-methods study. *Research in Developmental Disabilities*, *119*, 104104. <https://doi.org/10.1016/j.ridd.2021.104104>

Cardon, T., Wangsgard, N., & Dobson, N. (2019). Video Modeling Using Classroom Peers as Models to Increase Social Communication Skills in Children with ASD in an Integrated Preschool. *Education and Treatment of Children*, *42*(4), 515‑536. <https://doi.org/10.1353/etc.2019.0024>

Carpenter, B., Carpenter, J., Egerton, J., & Cockbill, B. (2016). The Engagement for Learning Framework : Connecting with learning and evidencing progress for children with autism spectrum conditions. *Advances in Autism*, *2*(1), 12‑23. <https://doi.org/10.1108/AIA-10-2015-0021>

Carr, E. G., & Blakeley-Smith, A. (2006). Classroom Intervention for Illness-Related Problem Behavior in Children With Developmental Disabilities. *Behavior Modification*, *30*(6), 901‑924. <https://doi.org/10.1177/0145445506290080>

Carter, E. W., Dykstra Steinbrenner, J. R., & Hall, L. J. (2019). Exploring Feasibility and Fit : Peer-Mediated Interventions for High School Students With Autism Spectrum Disorders. *School Psychology Review*, *48*(2), 157‑169. <https://doi.org/10.17105/SPR-2017-0112.V48-2>

Carter, E. W., Gustafson, J. R., Sreckovic, M. A., Dykstra Steinbrenner, J. R., Pierce, N. P., Bord, A., Stabel, A., Rogers, S., Czerw, A., & Mullins, T. (2017). Efficacy of Peer Support Interventions in General Education Classrooms for High School Students With Autism Spectrum Disorder. *Remedial and Special Education*, *38*(4), 207‑221. <https://doi.org/10.1177/0741932516672067>

Carter, M., Stephenson, J., Clark, T., Costley, D., Martin, J., Williams, K., Bruck, S., Davies, L., Browne, L., & Sweller, N. (2019). A comparison of two models of support for students with autism spectrum disorder in school and predictors of school success. *Research in Autism Spectrum Disorders*, *68*, 101452. <https://doi.org/10.1016/j.rasd.2019.101452>

Casey, S. D., & Merical, C. L. (2006). The Use of Functional Communication Training without Additional Treatment Procedures in an Inclusive School Setting. *Behavioral Disorders*, *32*(1), 46‑54. <https://doi.org/10.1177/019874290603200102>

Cassidy, C., Marwick, H., Deeney, L., & McLean, G. (2018). Philosophy with children, self-regulation and engaged participation for children with emotional-behavioural and social communication needs. *Emotional and Behavioural Difficulties*, *23*(1), 81‑96. <https://doi.org/10.1080/13632752.2017.1388654>

Causton-Theoharis, J., Ashby, C., & DeClouette, N. (2009). Relentless Optimism : Inclusive Postsecondary Opportunities for Students with Significant Disabilities. *Journal of Postsecondary Education and Disability*, *22*(2). <http://eric.ed.gov/ERICWebPortal/contentdelivery/servlet/ERICServlet?accno=EJ868134>

Chan, J. M., & O’Reilly, M. F. (2008). A Social Stories^tm^ Intervention Package For Students With Autism In Inclusive Classroom Settings. *Journal of Applied Behavior Analysis*, *41*(3), 405‑409. <https://doi.org/10.1901/jaba.2008.41-405>

Chan, J. M., O’Reilly, M. F., Lang, R. B., Boutot, E. A., White, P. J., Pierce, N., & Baker, S. (2011). Evaluation of a Social Stories^TM^ intervention implemented by pre-service teachers for students with autism in general education settings. *Research in  Spectrum Disorders*, *5*(2), 715‑721. <https://doi.org/10.1016/j.rasd.2010.08.005>

Chang, Y.-C., Shire, S. Y., Shih, W., Gelfand, C., & Kasari, C. (2016). Preschool Deployment of Evidence-Based Social Communication Intervention : JASPER in the Classroom. *Journal of Autism and Developmental Disorders*, *46*(6), 2211‑2223. <https://doi.org/10.1007/s10803-016-2752-2>

Chen, Y.-L., Martin, W., Vidiksis, R., & Patten, K. (2021). “A different environment for success:” a mixed-methods exploration of social participation outcomes among adolescents on the autism spectrum in an inclusive, interest-based school club. *International Journal of Developmental Disabilities*, *69*(5), 738‑747. <https://doi.org/10.1080/20473869.2021.2001729>

Chen, Y.-L., Murthi, K., Martin, W., Vidiksis, R., Riccio, A., & Patten, K. (2022). Experiences of Students, Teachers, and Parents Participating in an Inclusive, School-Based Informal Engineering Education Program. *Journal of Autism and Developmental Disorders*, *52*(8), 3574‑3585. <https://doi.org/10.1007/s10803-021-05230-2>

Cheung, P. P. P., Brown, T., Yu, M.-L., & Siu, A. M. H. (2021). The Effectiveness of a School-Based Social Cognitive Intervention on the Social Participation of Chinese Children with Autism. *Journal of autism and developmental disorders*, *51*(6), 1894‑1908. <https://doi.org/10.1007/s10803-020-04683-1>

Chou, Y.-C., & Park, H. R. (2021). Examining the moderating role of inclusiveness and social support in promoting social problem-solving among youth with autism spectrum disorder. *Education and Training in Autism and Developmental Disabilities*, *56*(3), 280‑292.

Chrysostomou, M., & Symeonidou, S. (2017). Education for disability equality through disabled people’s life stories and narratives : Working and learning together in a school-based professional development programme for inclusion. *European Journal of Special Needs Education*, *32*(4), 572‑585. <https://doi.org/10.1080/08856257.2017.1297574>

Chung, E. Y. (2020). Robot-Mediated Social Skill Intervention Programme for Children with Autism Spectrum Disorder : An ABA Time-Series Study. *International Journal of Social Robotics*, *13*(5), 1095‑1107. <https://doi.org/10.1007/s12369-020-00699-w>

Chung, Y.-C., & Douglas, K. H. (2015). A Peer Interaction Package for Students with Autism Spectrum Disorders who Use Speech-Generating Devices. *Journal of Developmental and Physical Disabilities*, *27*(6), 831‑849. <https://doi.org/10.1007/s10882-015-9461-1>

Cihak, D. F., Kildare, L. K., Smith, C. C., McMahon, D. D., & Quinn-Brown, L. (2012). Using Video Social Stories^TM^ to Increase Task Engagement for Middle School Students With Autism Spectrum Disorders. *Behavior Modification*, *36*(3), 399‑425. <https://doi.org/10.1177/0145445512442683>

Clemons, L. L., Mason, B. A., Garrison-Kane, L., & Wills, H. P. (2016). Self-Monitoring for High School Students With Disabilities : A Cross-Categorical Investigation of I-Connect. *Journal of Positive Behavior Interventions*, *18*(3), 145‑155. <https://doi.org/10.1177/1098300715596134>

Conroy, M. A., Boyd, B. A., Asmus, J. M., & Madera, D. (2007). A Functional Approach for Ameliorating Social Skills Deficits in Young Children With Autism Spectrum Disorders. *Infants & Young Children*, *20*(3), 242‑254. <https://doi.org/10.1097/01.IYC.0000277755.93313.7d>

Cook, A., Ogden, J., & Winstone, N. (2019). The impact of a school-based musical contact intervention on prosocial attitudes, emotions and behaviours : A pilot trial with autistic and neurotypical children. *Autism*, *23*(4), 933‑942. <https://doi.org/10.1177/1362361318787793>

Cook, A. (2020). Using an inclusive therapeutic theatre production to teach self-advocacy skills in young people with disabilities. *The Arts in Psychotherapy*, *71*, 101715. <https://doi.org/10.1016/j.aip.2020.101715>

D’Agostino, S., Douglas, S. N., & Horton, E. (2020). Inclusive Preschool Practitioners’ Implementation of Naturalistic Developmental Behavioral Intervention Using Telehealth Training. *Journal of autism and developmental disorders*, *50*(3), 864‑880. <https://doi.org/10.1007/s10803-019-04319-z>

Dean, M., Williams, J., Orlich, F., & Kasari, C. (2020). Adolescents with autism spectrum disorder and social skills groups at school : A randomized trial comparing intervention environment and peer composition. *School Psychology Review*, *49*(1), 60‑73. <https://doi.org/10.1080/2372966X.2020.1716636>

De Boer, A., Pijl, S. J., Minnaert, A., & Post, W. (2014). Evaluating the Effectiveness of an Intervention Program to Influence Attitudes of Students Towards Peers with Disabilities. *Journal of Autism and Developmental Disorders*, *44*(3), 572‑583. <https://doi.org/10.1007/s10803-013-1908-6>

Drmic, I. E., Aljunied, M., & Reaven, J. (2017). Feasibility, Acceptability and Preliminary Treatment Outcomes in a School-Based CBT Intervention Program for Adolescents with ASD and Anxiety in Singapore. *Journal of autism and developmental disorders*, *47*(12), 3909‑3929. <https://doi.org/10.1007/s10803-016-3007-y>

Dueñas, A. D., Plavnick, J. B., & Bak, M. Y. S. (2019). Effects of Joint Video Modeling on Unscripted Play Behavior of Children with Autism Spectrum Disorder. *Journal of Autism and Developmental Disorders*, *49*(1), 236‑247. <https://doi.org/10.1007/s10803-018-3719-2>

Dueñas, A. D., D’Agostino, S. R., & Plavnick, J. B. (2021). Teaching Young Children to Make Bids to Play to Peers With Autism Spectrum Disorder. *Focus on  and Other Developmental Disabilities*, *36*(4), 201‑212. <https://doi.org/10.1177/10883576211023326>

Dueñas, A. D., Wood, C., Maher, C. E., & Sanchula, G. (2022). Component analysis of a peer training program for teaching social responsiveness to neurotypical preschoolers. *Education & Treatment of Children*. <https://doi.org/10.1007/s43494-022-00073-7>

Eikeseth, S., Klintwall, L., Jahr, E., & Karlsson, P. (2012). Outcome for children with autism receiving early and intensive behavioral intervention in mainstream preschool and kindergarten settings. *Research in Autism Spectrum Disorders*, *6*(2), 829‑835. <https://doi.org/10.1016/j.rasd.2011.09.002>

Eldevik, S., Hastings, R. P., Jahr, E., & Hughes, J. C. (2012). Outcomes of behavioral intervention for children with autism in mainstream pre-school settings. *Journal of autism and developmental disorders*, *42*(2), 210‑220. <https://doi.org/10.1007/s10803-011-1234-9>

Fage, C., Consel, C., Etchegoyhen, K., Amestoy, A., Bouvard, M., Mazon, C., & Sauzéon, H. (2019). An emotion regulation app for school inclusion of children with ASD : Design principles and evaluation. *Computers & Education*, *131*, 1‑21. <https://doi.org/10.1016/j.compedu.2018.12.003>

Fage, C., Consel, C. Y., Balland, E., Etchegoyhen, K., Amestoy, A., Bouvard, M., & Sauzéon, H. (2018). Tablet Apps to Support First School Inclusion of Children With Autism Spectrum Disorders (ASD) in Mainstream Classrooms : A Pilot Study. *Frontiers in Psychology*, 9-20. <https://doi.org/10.3389/fpsyg.2018.02020>

Fees, B. S., Kaff, M., Holmberg, T., Teagarden, J., & Delreal, D. (2014). Children’s Responses to A Social Story Song in Three Inclusive Preschool Classrooms : A Pilot Study: Table 1. *Music Therapy Perspectives*, *32*(1), 71‑77. <https://doi.org/10.1093/mtp/miu007>

Feldman, E. K., & Matos, R. (2013). Training Paraprofessionals to Facilitate Social Interactions Between Children With Autism and Their Typically Developing Peers. *Journal of Positive Behavior Interventions*, *15*(3), 169‑179. <https://doi.org/10.1177/1098300712457421>

Feuerstein, J. L., & Landa, R. J. (2020). Implementation of Early Achievements for Childcare Providers : A cluster-randomized controlled trial. *Early Childhood Research Quarterly*, *53*, 520‑533. <https://doi.org/10.1016/j.ecresq.2020.06.006>

Fridell, A., Coco, C., Borg, A., & Bölte, S. (2023). School-based social skills group training (SKOLKONTAKT^TM^) : A pilot randomized controlled trial. *Frontiers in Psychology*, *14*, 128-288. <https://doi.org/10.3389/fpsyg.2023.1128288>

Fujiwara, A., & Sonoyama, S. (2019). Promoting social play based on ecological assessment and social play selection conditions of a child with autism spectrum disorder in an inclusive early childhood classroom. *Education and Training in Autism and Developmental Disabilities*, *54*(3), 288‑300.

Gann, C. J., Ferro, J. B., Umbreit, J., & Liaupsin, C. J. (2014). Effects of a Comprehensive Function-Based Intervention Applied Across Multiple Educational Settings. *Remedial and Special Education*, *35*(1), 50‑60. <https://doi.org/10.1177/0741932513501088>

Gena, A. (2006). The effects of prompting and social reinforcement on establishing social interactions with peers during the inclusion of four children with autism in preschool. *International Journal of Psychology*, *41*(6), 541‑554. <https://doi.org/10.1080/00207590500492658>

Gengoux, G. W. (2015). Priming for Social Activities : Effects on Interactions Between Children With Autism and Typically Developing Peers. *Journal of Positive Behavior Interventions*, *17*(3), 181‑192. <https://doi.org/10.1177/1098300714561862>

Giannopoulou, I., Pasalari, E., Korkoliakou, P., & Douzenis, A. (2019). Raising Autism Awareness among Greek Teachers. *International Journal of Disability, Development and Education*, *66*(1), 70‑81. <https://doi.org/10.1080/1034912X.2018.1462474>

Goldingay, S., Stagnitti, K., Robertson, N., Pepin, G., Sheppard, L., & Dean, B. (2020). Implicit play or explicit cognitive behaviour therapy : The impact of intervention approaches to facilitate social skills development in adolescents. *Australian Occupational Therapy Journal*, *67*(4), 360‑372. <https://doi.org/10.1111/1440-1630.12673>

Grey, I. M., Bruton, C., Honan, R., McGuinness, R., & Daly, M. (2007). Co‐operative Learning for Children with an Autistic Spectrum Disorder (ASD) in Mainstream and Special Class Settings : An exploratory study. *Educational Psychology in Practice*, *23*(4), 317‑327. <https://doi.org/10.1080/02667360701660936>

Grindle, C. F., Hastings, R. P., Saville, M., Carl Hughes, J., Kovshoff, H., & Huxley, K. (2009). Integrating evidence-based behavioural teaching methods into education for children with autism. *Educational and Child Psychology*, *26*(4), 65‑81. <https://doi.org/10.53841/bpsecp.2009.26.4.65>

Grindle, C. F., Hastings, R. P., Saville, M., Hughes, J. C., Huxley, K., Kovshoff, H., Griffith, G. M., Walker-Jones, E., Devonshire, K., & Remington, B. (2012). Outcomes of a Behavioral Education Model for Children With Autism in a Mainstream School Setting. *Behavior Modification*, *36*(3), 298‑319. <https://doi.org/10.1177/0145445512441199>

Guralnick, M. J., Connor, R. T., Neville, B., & Hammond, M. A. (2006). Promoting the Peer-Related Social Development of Young Children With Mild Developmental Delays : Effectiveness of a Comprehensive Intervention. *American Journal on Mental Retardation*, *111*(5), 336. [https://doi.org/10.1352/0895-8017(2006)111[336:PTPSDO]2.0.CO;2](https://doi.org/10.1352/0895-8017(2006)111%5b336:PTPSDO%5d2.0.CO;2)

Haas, K., Carter, M., Stephenson, J., & Gibbs, V. (2022). Benefits and Challenges of a Hybrid Distance Education Program for Autistic School-Age Students : Parent, Student and Teacher Perspectives. *Australasian Journal of Special and Inclusive Education*, *46*(1), 61‑73. <https://doi.org/10.1017/jsi.2022.2>

Hajri, M., Abbes, Z., Ben Yahia, H., Boudali, M., Bouden, A., Mrabet, A., & Amado, I. (2019). Remédiation cognitive et fonctionnement scolaire chez les enfants avec trouble du spectre autistique. *Neuropsychiatrie de l’Enfance et de l’Adolescence*, *67*(1), 19‑24. <https://doi.org/10.1016/j.neurenf.2018.11.001>

Haley, J. L., Heick, P. F., & Luiselli, J. K. (2010). Use of an Antecedent Intervention to Decrease Vocal Stereotypy of a Student With Autism in the General Education Classroom. *Child & Family Behavior Therapy*, *32*(4), 311‑321. <https://doi.org/10.1080/07317107.2010.515527>

Hampshire, P. K., & Crawford, M. (2022). Expanding toy play through visually structured tasks and scripting for young children with developmental disabilities. *Journal of Research in Special Educational Needs*, *22*(1), 44‑53. <https://doi.org/10.1111/1471-3802.12526>

Hansen, S. G., Raulston, T. J., Machalicek, W., Frantz, R., Drew, C., Erturk, B., & Squires, J. (2019). Peer-Mediated Joint Attention Intervention in the Preschool Classroom. *The Journal of Special Education*, *53*(2), 96‑107. <https://doi.org/10.1177/0022466918807464>

Harjusola-Webb, S. M., & Robbins, S. H. (2012). The Effects of Teacher-Implemented Naturalistic Intervention on the Communication of Preschoolers With Autism. *Topics in Early Childhood Special Education*, *32*(2), 99‑110. <https://doi.org/10.1177/0271121410397060>

Harper, C. B., Symon, J. B. G., & Frea, W. D. (2008). Recess is Time-in : Using Peers to Improve Social Skills of Children with Autism. *Journal of Autism and Developmental Disorders*, *38*(5), 815‑826. <https://doi.org/10.1007/s10803-007-0449-2>

Hartzell, R., Gann, C., Liaupsin, C., & Clem, S. (2015). Increasing social engagement in an inclusive environment. *Education and Training in Autism and Developmental Disabilities*, *50*(3), 264‑277.

Hodges, A., Cordier, R., Joosten, A., Bourke-Taylor, H., & Chen, Y.-W. (2022). Evaluating the feasibility, fidelity, and preliminary effectiveness of a school-based intervention to improve the school participation and feelings of connectedness of elementary school students on the autism spectrum. *PLOS ONE*, *17*(6), e0269098. <https://doi.org/10.1371/journal.pone.0269098>

Hu, X., & Lee, G. T. (2020). Improving Emotional Skills for Chinese Elementary Children with Autism Spectrum Disorder. *Child & Family Behavior Therapy*, *42*(4), 231‑257. <https://doi.org/10.1080/07317107.2020.1809192>

Hu, X., & Han, Z. R. (2019). Effects of gesture-based match-to-sample instruction via virtual reality technology for Chinese students with autism spectrum disorders. *International Journal of Developmental Disabilities*, *65*(5), 327‑336. <https://doi.org/10.1080/20473869.2019.1602350>

Hu, X., Zheng, Q., & Lee, G. T. (2018). Using Peer-Mediated LEGO® Play Intervention to Improve Social Interactions for Chinese Children with Autism in an Inclusive Setting. *Journal of Autism and Developmental Disorders*, *48*(7), 2444‑2457. <https://doi.org/10.1007/s10803-018-3502-4>

Huber, H. B., Carter, E. W., Lopano, S. E., & Stankiewicz, K. C. (2018). Using Structural Analysis to Inform Peer Support Arrangements for High School Students With Severe Disabilities. *American Journal on Intellectual and Developmental Disabilities*, *123*(2), 119‑139. <https://doi.org/10.1352/1944-7558-123.2.119>

Hundert, J., Rowe, S., & Harrison, E. (2014). The Combined Effects of Social Script Training and Peer Buddies on Generalized Peer Interaction of Children With ASD in Inclusive Classrooms. *Focus on Autism and Other Developmental Disabilities*, *29*(4), 206‑215. <https://doi.org/10.1177/1088357614522288>

Iadarola, S., Shih, W., Dean, M., Blanch, E., Harwood, R., Hetherington, S., Mandell, D., Kasari, C., & Smith, T. (2018). Implementing a Manualized, Classroom Transition Intervention for Students With ASD in Underresourced Schools. *Behavior Modification*, *42*(1), 126‑147. <https://doi.org/10.1177/0145445517711437>

Imasaka, T., Lee, P. L., Anderson, A., Wong, C. W. R., Moore, D. W., Furlonger, B., & Bussaca, M. (2020). Improving Compliance in Primary School Students with Autism Spectrum Disorder. *Journal of Behavioral Education*, *29*(4), 763‑786. <https://doi.org/10.1007/s10864-019-09346-5>

Ip, H. H. S., Wong, S. W. L., Chan, D. F. Y., Li, C., Kon, L. L., Ma, P. K., Lau, K. S. Y., & Byrne, J. (2022). Enhance affective expression and social reciprocity for children with autism spectrum disorder : Using virtual reality headsets at schools. *Interactive Learning Environments*, 1‑24. <https://doi.org/10.1080/10494820.2022.2107681>

Jameson, J. M., Walker, R., Utley, K., & Maughan, R. (2012). A Comparison of Embedded Total Task Instruction in Teaching Behavioral Chains to Massed One-on-One Instruction for Students With Intellectual Disabilities : Accessing General Education Settings and Core Academic Content. *Behavior Modification*, *36*(3), 320‑340. <https://doi.org/10.1177/0145445512440574>

Jones, K., & Howley, M. (2010). An investigation into an interaction programme for children on the autism spectrum : Outcomes for children, perceptions of schools and a model for training: An Investigation into an Interaction Programme for Children on the Autism Spectrum. *Journal of Research in Special Educational Needs*, *10*(2), 115‑123. <https://doi.org/10.1111/j.1471-3802.2010.01153.x>

Karanth, P., & Chandhok, T. S. (2013). Impact of Early Intervention on Children with Autism Spectrum Disorders as Measured by Inclusion and Retention in Mainstream Schools. *The Indian Journal of Pediatrics*, *80*(11), 911‑919. <https://doi.org/10.1007/s12098-013-1014-y>

Kasari, C., Dean, M., Kretzmann, M., Shih, W., Orlich, F., Whitney, R., Landa, R., Lord, C., & King, B. (2016). Children with autism spectrum disorder and social skills groups at school : A randomized trial comparing intervention approach and peer composition. *Journal of Child Psychology and Psychiatry*, *57*(2), 171‑179. <https://doi.org/10.1111/jcpp.12460>

Kasari, C., Rotheram‐Fuller, E., Locke, J., & Gulsrud, A. (2012). Making the connection : Randomized controlled trial of social skills at school for children with autism spectrum disorders. *Journal of Child Psychology and Psychiatry*, *53*(4), 431‑439. <https://doi.org/10.1111/j.1469-7610.2011.02493.x>

Kemp, S., Petriwskyj, A., Shakespeare-Finch, J., & Thorpe, K. (2013). What if you’re really different ? Case studies of children with high functioning Autism participating in the get REAL programme who had atypical learning trajectories. *European Journal of Special Needs Education*, *28*(1), 91‑108. <https://doi.org/10.1080/08856257.2012.749609>

Kim, S., Koegel, R. L., & Koegel, L. K. (2017). Training Paraprofessionals to Target Socialization in Students With ASD : Fidelity of Implementation and Social Validity. *Journal of Positive Behavior Interventions*, *19*(2), 102‑114. <https://doi.org/10.1177/1098300716669813>

Kisbu-Sakarya, Y., & Doenyas, C. (2021). Can school teachers’ willingness to teach ASD-inclusion classes be increased via special education training? Uncovering mediating mechanisms. *Research in Developmental Disabilities*, *113*, 103941. <https://doi.org/10.1016/j.ridd.2021.103941>

Knight, V. F., Kuntz, E. M., & Brown, M. (2018). Paraprofessional-Delivered Video Prompting to Teach Academics to Students with Severe Disabilities in Inclusive Settings. *Journal of Autism and Developmental Disorders*, *48*(6), 2203‑2216. <https://doi.org/10.1007/s10803-018-3476-2>

Koegel, L. K., Kuriakose, S., Singh, A. K., & Koegel, R. L. (2012). Improving Generalization of Peer Socialization Gains in Inclusive School Settings Using Initiations Training. *Behavior Modification*, *36*(3), 361‑377. <https://doi.org/10.1177/0145445512445609>

Koegel, L. K., Vernon, T. W., Koegel, R. L., Koegel, B. L., & Paullin, A. W. (2012). Improving Social Engagement and Initiations Between Children With Autism Spectrum Disorder and Their Peers in Inclusive Settings. *Journal of Positive Behavior Interventions*, *14*(4), 220‑227. <https://doi.org/10.1177/1098300712437042>

Koegel, R., Kim, S., Koegel, L., & Schwartzman, B. (2013). Improving Socialization for High School Students with ASD by Using Their Preferred Interests. *Journal of Autism and Developmental Disorders*, *43*(9), 2121‑2134. <https://doi.org/10.1007/s10803-013-1765-3>

Koegel, R. L., Kim, S., & Koegel, L. K. (2014). Training Paraprofessionals to Improve Socialization in Students with ASD. *Journal of Autism and Developmental Disorders*, *44*(9), 2197‑2208. <https://doi.org/10.1007/s10803-014-2094-x>

Koenig, K. P., Feldman, J. M., Siegel, D., Cohen, S., & Bleiweiss, J. (2014). Issues in Implementing a Comprehensive Intervention for Public School Children With Autism Spectrum Disorders. *Journal of Prevention & Intervention in the Community*, *42*(4), 248‑263. <https://doi.org/10.1080/10852352.2014.943638>

Kotsopoulos, S. I., Karaivazoglou, K., Florou, I. S., Gyftogianni, M. I., Papadaki, E. J., & Kotsopoulou, A. (2021). Systematic Intervention for Children with Autism Spectrum Disorder and Integration in Regular School Classes : A Naturalistic Study. *Global Pediatric Health*, *8*, 233-379. <https://doi.org/10.1177/2333794X211012988>

Kretzmann, M., Shih, W., & Kasari, C. (2015). Improving Peer Engagement of Children With Autism on the School Playground : A Randomized Controlled Trial. *Behavior Therapy*, *46*(1), 20‑28. <https://doi.org/10.1016/j.beth.2014.03.006>

Krier, J., & Lambros, K. M. (2021). Increasing joint attention and social play through peer‐mediated intervention : A single case design. *Psychology in the Schools*, *58*(3), 494‑514. <https://doi.org/10.1002/pits.22460>

Kwon, E. Y., Cannon, J. E., Knight, V. F., Mercer, S. H., & Guardino, C. (2023). Effects of Social Stories on Increasing Social Interaction and Engagement of Deaf and Hard of Hearing Students with Autism Spectrum Disorder in Inclusive Settings. *Journal of Autism and Developmental Disorders*, *53*(5), 1915‑1929. <https://doi.org/10.1007/s10803-022-05430-4>

Lam, S., Tsang, N., Keung, Y.-C., Tong, Y., Mok, F., Chiu, A., Lai, Y., Yuen, L., & Soh, D. (2019). A comprehensive service delivery model for preschoolers with special educational needs : Its characteristics and effectiveness. *Research in Developmental Disabilities*, *85*, 20‑30. <https://doi.org/10.1016/j.ridd.2018.10.005>

Lambert, R., Sugita, T., Yeh, C., Hunt, J. H., & Brophy, S. (2020). Documenting increased participation of a student with autism in the standards for mathematical practice. *Journal of Educational Psychology*, *112*(3), 494‑513. <https://doi.org/10.1037/edu0000425>

Landor, F., & Perepa, P. (2017). Do resource bases enable social inclusion of students with Asperger syndrome in a mainstream secondary school? *Support for Learning*, *32*(2), 129‑143. <https://doi.org/10.1111/1467-9604.12158>

Leão, A. T., Camargo, S. P. H., & Frison, L. M. B. (2019). Communication of students with ASD : A self-regulation of learning based intervention. *Psicologia - Teoria e Prática*, *21*(3). <https://doi.org/10.5935/1980-6906/psicologia.v21n3p473-500>

Ledbetter-Cho, K., Lang, R., Moore, M., Davenport, K., Murphy, C., Lee, A., O’Reilly, M., & Watkins, L. (2017). Effects of video-enhanced activity schedules on academic skills and collateral behaviors in children with autism. *International Journal of Developmental Disabilities*, *63*(4), 228‑237. <https://doi.org/10.1080/20473869.2017.1290022>

Ledford, J. R., & Wehby, J. H. (2015). Teaching Children with Autism in Small Groups with Students Who are At-Risk for Academic Problems : Effects on Academic and Social Behaviors. *Journal of Autism and Developmental Disorders*, *45*(6), 1624‑1635. <https://doi.org/10.1007/s10803-014-2317-1>

Ledford, J. R., Zimmerman, K. N., Chazin, K. T., Patel, N. M., Morales, V. A., & Bennett, B. P. (2017). Coaching Paraprofessionals to Promote Engagement and Social Interactions During Small Group Activities. *Journal of Behavioral Education*, *26*(4), 410‑432. <https://doi.org/10.1007/s10864-017-9273-8>

Lee, K. Y. S., Crooke, P. J., Lui, A. L. Y., Kan, P. P. K., Mark, Y., Van Hasselt, C. A., & Tong, M. C. F. (2016). The Outcome of a Social Cognitive Training for Mainstream Adolescents with Social Communication Deficits in a Chinese Community. *International Journal of Disability, Development and Education*, *63*(2), 201‑223. <https://doi.org/10.1080/1034912X.2015.1065960>

Lehane, P., & Senior, J. (2020). Collaborative teaching : Exploring the impact of co-teaching practices on the numeracy attainment of pupils with and without special educational needs. *European Journal of Special Needs Education*, *35*(3), 303‑317. <https://doi.org/10.1080/08856257.2019.1652439>

Leifler, E., Coco, C., Fridell, A., Borg, A., & Bölte, S. (2022). Social Skills Group Training for Students with Neurodevelopmental Disabilities in Senior High School—A Qualitative Multi-Perspective Study of Social Validity. *International Journal of Environmental Research and Public Health*, *19*(3), 1487. <https://doi.org/10.3390/ijerph19031487>

Levy, J., & Dunsmuir, S. (2020). Lego Therapy : Building social skills for adolescents with an autism spectrum disorder. *Educational and Child Psychology*, *37*(1), 58‑83. <https://doi.org/10.53841/bpsecp.2020.37.1.58>

Locke, J., Kang-Yi, C., Frederick, L., & Mandell, D. S. (2020). Individual and organizational characteristics predicting intervention use for children with autism in schools. *Autism*, *24*(5), 1152‑1163. <https://doi.org/10.1177/1362361319895923>

Locke, J., Kang‐Yi, C., Pellecchia, M., & Mandell, D. S. (2019). It’s messy but real : A pilot study of the implementation of a social engagement intervention for children with autism in schools. *Journal of Research in Special Educational Needs*, *19*(2), 135‑144. <https://doi.org/10.1111/1471-3802.12436>

Locke, J., Olsen, A., Wideman, R., Downey, M. M., Kretzmann, M., Kasari, C., & Mandell, D. S. (2015). A Tangled Web : The Challenges of Implementing an Evidence-Based Social Engagement Intervention for Children With Autism in Urban Public School Settings. *Behavior Therapy*, *46*(1), 54‑67. <https://doi.org/10.1016/j.beth.2014.05.001>

Locke, J., Rotheram-Fuller, E., Harker, C., Kasari, C., & Mandell, D. S. (2019). Comparing a Practice-Based Model with a Research-Based Model of social skills interventions for children with autism in schools. *Research in Autism Spectrum Disorders*, *62*, 10‑17. <https://doi.org/10.1016/j.rasd.2019.02.002>

Locke, J., Shih, W., Kang-Yi, C. D., Caramanico, J., Shingledecker, T., Gibson, J., Frederick, L., & Mandell, D. S. (2019). The impact of implementation support on the use of a social engagement intervention for children with autism in public schools. *Autism*, *23*(4), 834‑845. <https://doi.org/10.1177/1362361318787802>

Locke, J., Wolk, C. B., Harker, C., Olsen, A., Shingledecker, T., Barg, F., Mandell, D., & Beidas, R. (2017). Pebbles, rocks, and boulders : The implementation of a school-based social engagement intervention for children with autism. *Autism*, *21*(8), 985‑994. <https://doi.org/10.1177/1362361316664474>

López, J. M., Moreno-Rodríguez, R., Alcover, C.-M., Garrote, I., & Sánchez, S. (2017). Effects of a Program of Sport Schools on Development of Social and Psychomotor Skills of People with Autistic Spectrum Disorders : A Pilot Project. *Journal of Education and Training Studies*, *5*(8), 167. <https://doi.org/10.11114/jets.v5i8.2555>

Lorenzo, G., Pomares, J., & Lledó, A. (2013). Inclusion of immersive virtual learning environments and visual control systems to support the learning of students with Asperger syndrome. *Computers & Education*, *62*, 88‑101. <https://doi.org/10.1016/j.compedu.2012.10.028>

Low, H. M., Wong, T. P., Lee, L. W., Makesavanh, S., Vongsouangtham, B., Phannalath, V., Che Ahmad, A., & Lee, A. S. S. (2021). Can pictorial narration offer a solution to teacher training on the effective inclusion of students with autism spectrum disorder in low-resource settings? Investigation on knowledge and stigma change. *Autism*, *25*(5), 1216‑1233. <https://doi.org/10.1177/1362361320984899>

Macdonald, L., Trembath, D., Ashburner, J., Costley, D., & Keen, D. (2018). The use of visual schedules and work systems to increase the on‐task behaviour of students on the autism spectrum in mainstream classrooms. *Journal of Research in Special Educational Needs*, *18*(4), 254‑266. <https://doi.org/10.1111/1471-3802.12409>

Macoun, S. J., Schneider, I., Bedir, B., Sheehan, J., & Sung, A. (2021). Pilot Study of an Attention and Executive Function Cognitive Intervention in Children with Autism Spectrum Disorders. *Journal of Autism and Developmental Disorders*, *51*(8), 2600‑2610. <https://doi.org/10.1007/s10803-020-04723-w>

Mandy, W., Murin, M., Baykaner, O., Staunton, S., Cobb, R., Hellriegel, J., Anderson, S., & Skuse, D. (2016). Easing the transition to secondary education for children with autism spectrum disorder : An evaluation of the Systemic Transition in Education Programme for Autism Spectrum Disorder (STEP-ASD). *Autism: the international journal of research and practice*, *20*(5), 580‑590. <https://doi.org/10.1177/1362361315598892>

Masi, A., Azim, S. I., Khan, F., Karlov, L., & Eapen, V. (2022). Dissemination of Early Intervention Program for Preschool Children on the Autism Spectrum into Community Settings : An Evaluation. *International Journal of Environmental Research and Public Health*, *19*(5), 2555. <https://doi.org/10.3390/ijerph19052555>

Maye, M., Sanchez, V. E., Stone-MacDonald, A., & Carter, A. S. (2020). Early Interventionists’ Appraisals of Intervention Strategies for Toddlers with Autism Spectrum Disorder and Their Peers in Inclusive Childcare Classrooms. *Journal of Autism and Developmental Disorders*, *50*(11), 4199‑4208. <https://doi.org/10.1007/s10803-020-04456-w>

McAllister, K., & Maguire, B. (2012). A design model : The A utism S pectrum D isorder C lassroom D esign K it. *British Journal of Special Education*, *39*(4), 201‑208. <https://doi.org/10.1111/1467-8578.12006>

McCurdy, E. E., & Cole, C. L. (2014). Use of a Peer Support Intervention for Promoting Academic Engagement of Students with Autism in General Education Settings. *Journal of Autism and Developmental Disorders*, *44*(4), 883‑893. <https://doi.org/10.1007/s10803-013-1941-5>

McDaniel, S. L., Hall, L. J., & Kraemer, B. K. (2022). Exploring Extracurricular Clubs for Building Social Competence of Students With Autism. *Frontiers in Psychiatry*, *13*, 840-1294. <https://doi.org/10.3389/fpsyt.2022.840294>

Morrier, M. J., McGee, G. G., & Daly, T. (2009). Effects of toy selection and arrangement on the social behaviors of an inclusive group of preschool-aged children with and without autism. *Early Childhood Services: An Interdisciplinary Journal of Effectiveness*, *3*(2), 157‑177.

Morris, S., O’Reilly, G., & Byrne, M. K. (2020). Understanding Our Peers with Pablo : Exploring the Merit of an Autism Spectrum Disorder De-stigmatisation Programme Targeting Peers in Irish Early Education Mainstream Settings. *Journal of Autism and Developmental Disorders*, *50*(12), 4385‑4400. <https://doi.org/10.1007/s10803-020-04464-w>

Mpella, M., Evaggelinou, C., Koidou, E., & Tsigilis, N. (2019). The Effects of a Theatrical Play Programme on Social Skills Development for Young Children with Autism Spectrum Disorders. *International Journal of Special Education*, *33*(4). <http://eric.ed.gov/ERICWebPortal/contentdelivery/servlet/ERICServlet?accno=EJ1219307>

Mukkiri, S., Kandasamy, P., Subramanian, M., Chandrasekaran, V., & Kattimani, S. (2022). Content validation of school readiness module and school readiness scale for assessing school readiness in children with autism spectrum disorder. *Asian Journal of Psychiatry*, *71*, 73-103. <https://doi.org/10.1016/j.ajp.2022.103073>

Nakutin, S. N., & Gutierrez, G. (2019). Effect of Physical Activity on Academic Engagement and Executive Functioning in Children With ASD. *School Psychology Review*, *48*(2), 177‑184. <https://doi.org/10.17105/SPR-2017-0124.V48-2>

Nelson, C., McDonnell, A. P., Johnston, S. S., Crompton, A., & Nelson, A. R. (2007). Keys to play : A strategy to increase the social interactions of young children with autism and their typically developing peers. *Education and Training in Developmental Disabilities*, *42*(2), 165‑181.

Nelson, C., Paul, K., Johnston, S. S., & Kidder, J. E. (2017). Use of a creative dance intervention package to increase social engagement and play complexity of young children with autism spectrum disorder. *Education and Training in Autism and Developmental Disabilities*, *52*(2), 170‑185.

Normand, C. L., & Moreau, A. C. (2009). Early intervention in daycare settings for children with pervasive developmental disorders: Impacts on children and their parents. *Revue francophone de la déficience intellectuelle*, 20, 56-64.

O’Connor, A. B., & Healy, O. (2010). Long-term post-intensive behavioral intervention outcomes for five children with Autism Spectrum Disorder. *Research in Autism Spectrum Disorders*, *4*(4), 594‑604. <https://doi.org/10.1016/j.rasd.2009.12.002>

O’Connor, E. (2016). The use of ‘Circle of Friends’ strategy to improve social interactions and social acceptance : A case study of a child with Asperger’s Syndrome and other associated needs. *Support for Learning*, *31*(2), 138‑147. <https://doi.org/10.1111/1467-9604.12122>

O’Hagan, B., Sonikar, P., Grace, R., Castillo, D., Chen, E., Agrawal, M., Dufresne, S., Rossetti, Z., Bartolotti, L., & Krauss, S. (2022). Youth and caregivers’ perspective on teens engaged as mentors (team) : An inclusive peer mentoring program for autistic adolescents. *Journal of Autism and Developmental Disorders*. <https://doi.org/10.1007/s10803-022-05543-w>

Odluyurt, S. (2013). A Comparison of the Effects of Direct Modeling and Video Modeling Provided by Peers to Students with Autism Who Are Attending in Rural Play Teaching in an Inclusive Setting. *Educational Sciences: Theory and Practice*, *13*(1). <http://eric.ed.gov/ERICWebPortal/contentdelivery/servlet/ERICServlet?accno=EJ1016663>

Odluyurt, S., Tekin-Iftar, E., & Ersoy, G. (2014). Effects of school counselor supervised peer tutoring in inclusive settings on meeting IEP outcomes of students with developmental disabilities. *Education and Training in Autism and Developmental Disabilities*, *49*(3), 415‑428.

O’Haire, M. E., McKenzie, S. J., Beck, A. M., & Slaughter, V. (2013). Social Behaviors Increase in Children with Autism in the Presence of Animals Compared to Toys. *PLoS ONE*, *8*(2), 57010. <https://doi.org/10.1371/journal.pone.0057010>

Öhlböck, E., Stinson, M., McClintock, K., & Turtle, B. (2023). Evaluating the effectiveness of key components of Zones of Regulation^TM^ curriculum training on teachers’ self‐efficacy at managing self‐regulation needs in autistic pupils. *British Journal of Special Education*, 12501-1467. <https://doi.org/10.1111/1467-8578.12501>

Ojea, M. R. (2018). Contributions of neurological psychology and social theory to facilitate the learning of children with autistic spectrum disorders through a specific program of development of conceptual categories. *International Journal of Developmental Disabilities*, *64*(4‑5), 230‑237. <https://doi.org/10.1080/20473869.2017.1326685>

Oka, T., Ishikawa, S., Saito, A., Maruo, K., Stickley, A., Watanabe, N., Sasamori, H., Shioiri, T., & Kamio, Y. (2021). Changes in self-efficacy in Japanese school-age children with and without high autistic traits after the Universal Unified Prevention Program : A single-group pilot study. *Child and Adolescent Psychiatry and Mental Health*, *15*(1), 42. <https://doi.org/10.1186/s13034-021-00398-y>

Olivar-Parra, J.-S., De-La-Iglesia-Gutiérrez, M., & Forns, M. (2011). Training Referential Communicative Skills to Individuals with Autism Spectrum Disorder : A Pilot Study. *Psychological Reports*, *109*(3), 921‑939. <https://doi.org/10.2466/10.11.15.28.PR0.109.6.921-939>

Ostmeyer, K., & Scarpa, A. (2012). Examining School‐Based Social Skills Program Needs and Barriers for Students with High‐Functioning Autism Spectrum Disorders Using Participatory Action Research. *Psychology in the Schools*, *49*(10), 932‑941. <https://doi.org/10.1002/pits.21646>

Owen-DeSchryver, J. S., Carr, E. G., Cale, S. I., & Blakeley-Smith, A. (2008). Promoting Social Interactions Between Students With Autism Spectrum Disorders and Their Peers in Inclusive School Settings. *Focus on Autism and Other Developmental Disabilities*, *23*(1), 15‑28. <https://doi.org/10.1177/1088357608314370>

Özerk, M., & Özerk, K. (2015). A Bilingual Child Learns Social Communication Skills through Video Modeling—A Single Case Study in a Norwegian School Setting. *International Electronic Journal of Elementary Education*, *8*(1). <http://eric.ed.gov/ERICWebPortal/contentdelivery/servlet/ERICServlet?accno=EJ1078781>

Panerai, S., Zingale, M., Trubia, G., Finocchiaro, M., Zuccarello, R., Ferri, R., & Elia, M. (2009). Special Education Versus Inclusive Education : The Role of the TEACCH Program. *Journal of Autism and Developmental Disorders*, *39*(6), 874‑882. <https://doi.org/10.1007/s10803-009-0696-5>

Panganiban, J. L., Shire, S. Y., Williams, J., & Kasari, C. (2022). Supporting peer engagement for low-income preschool students with autism spectrum disorder during academic instruction : A pilot randomized trial. *Autism*, *26*(8), 2175‑2187. <https://doi.org/10.1177/13623613221085339>

Parsons, L., Cordier, R., Munro, N., & Joosten, A. (2019). A Randomized Controlled Trial of a Play-Based, Peer-Mediated Pragmatic Language Intervention for Children With Autism. *Frontiers in Psychology*, *10*, 1960. <https://doi.org/10.3389/fpsyg.2019.01960>

Pickard, K., Meyer, A., Reyes, N., Tanda, T., & Reaven, J. (2022). Using evaluative frameworks to examine the implementation outcomes of a cognitive behavioral therapy program for autistic students with anxiety within public school settings. *Autism*, *26*(3), 640‑653. <https://doi.org/10.1177/13623613211065797>

Pingale, V., Fletcher, T., & Candler, C. (2019). The Effects of Sensory Diets on Children’s Classroom Behaviors. *Journal of Occupational Therapy, Schools, & Early Intervention*, *12*(2), 225‑238. <https://doi.org/10.1080/19411243.2019.1592054>

Pokorski, E. A., Barton, E. E., Ledford, J. R., Taylor, A. L., Johnson, E., & Winters, H. K. (2019). Comparison of Antecedent Activities for Increasing Engagement in a Preschool Child with ASD during a Small Group Activity. *Education and Training in Autism and Developmental Disabilities*, *54*(1).

Radley, K. C., Ford, W. B., Battaglia, A. A., & McHugh, M. B. (2014). The Effects of a Social Skills Training Package on Social Engagement of Children With Autism Spectrum Disorders in a Generalized Recess Setting. *Focus on Autism and Other Developmental Disabilities*, *29*(4), 216‑229. <https://doi.org/10.1177/1088357614525660>

Radley, K. C., McHugh, M. B., Taber, T., Battaglia, A. A., & Ford, W. B. (2017). School-Based Social Skills Training for Children With Autism Spectrum Disorder. *Focus on Autism and Other Developmental Disabilities*, *32*(4), 256‑268. <https://doi.org/10.1177/1088357615583470>

Ranson, N. J., & Byrne, M. K. (2014). Promoting Peer Acceptance of Females with Higher-functioning Autism in a Mainstream Education Setting : A Replication and Extension of the Effects of an Autism Anti-Stigma Program. *Journal of Autism and Developmental Disorders*, *44*(11), 2778‑2796. <https://doi.org/10.1007/s10803-014-2139-1>

Ratcliffe, B., Wong, M., Dossetor, D., & Hayes, S. (2014). Teaching social–emotional skills to school-aged children with Autism Spectrum Disorder : A treatment versus control trial in 41 mainstream schools. *Research in Autism Spectrum Disorders*, *8*(12), 1722‑1733. <https://doi.org/10.1016/j.rasd.2014.09.010>

Rayner, C., & Fluck, A. (2014). Pre-service teachers’ perceptions of simSchool as preparation for inclusive education : A pilot study. *Asia-Pacific Journal of Teacher Education*, *42*(3), 212‑227. <https://doi.org/10.1080/1359866X.2014.927825>

Reaven, J., Pickard, K., Meyer, A. T., Hayutin, L., Middleton, C., Reyes, N. M., Tanda, T., Stahmer, A., Blakeley-Smith, A., & Boles, R. E. (2024). Implementing school-based cognitive behavior therapy for anxiety in students with autism or suspected autism via a train-the-trainer approach : Results from a clustered randomized trial. *Autism*, *28*(2), 484‑497. <https://doi.org/10.1177/13623613231175951>

Reed, S. (2019). Embedding effective autism knowledge and practice in mainstream primary schools : The development of a successful model for outreach. *Support for Learning*, *34*(3), 242‑253. <https://doi.org/10.1111/1467-9604.12264>

Reeves, L. M., Umbreit, J., Ferro, J. B., & Liaupsin, C. J. (2013). Function-based intervention to support the inclusion of students with autism. *Education and Training in Autism and Developmental Disabilities*, *48*(3), 379‑391.

Reiter, S., & Vitani, T. (2007). Inclusion of pupils with autism : The effect of an intervention program on the regular pupils’ burnout, attitudes and quality of mediation. *Autism*, *11*(4), 321‑333. <https://doi.org/10.1177/1362361307078130>

Richard, V., & Goupil, G. (2007). Les groupes de jeux intégrés destinés aux enfants ayant un trouble envahissant du développement : Modalités de participation et perceptions des pairs de classes ordinaires. *Revue de psychoéducation*, *36*(1), 109‑127. <https://doi.org/10.7202/1097198ar>

Roberts, G. J., Mize, M., Reutebuch, C. K., Falcomata, T., Capin, P., & Steelman, B. L. (2019). Effects of a Self-Management with Peer Training Intervention on Academic Engagement for High School Students with Autism Spectrum Disorder. *Journal of Behavioral Education*, *28*(4), 456‑478. <https://doi.org/10.1007/s10864-018-09317-2>

Robinson, J., Gershwin, T., & London, D. (2019). Maintaining Safety and Facilitating Inclusion : Using Applied Behavior Analysis to Address Self-Injurious Behaviors Within General Education Classrooms. *Beyond Behavior*, *28*(3), 154‑167. <https://doi.org/10.1177/1074295619870473>

Robinson, S. E. (2011). Teaching Paraprofessionals of Students With Autism to Implement Pivotal Response Treatment in Inclusive School Settings Using a Brief Video Feedback Training Package. *Focus on Autism and Other Developmental Disabilities*, *26*(2), 105‑118. <https://doi.org/10.1177/1088357611407063>

Rodríguez-Medina, J., Martín-Antón, L. J., Carbonero, M. A., & Ovejero, A. (2016). Peer-Mediated Intervention for the Development of Social Interaction Skills in High-Functioning Autism Spectrum Disorder : A Pilot Study. *Frontiers in Psychology*, *7*. <https://doi.org/10.3389/fpsyg.2016.01986>

Rosenbloom, R., Mason, R. A., Wills, H. P., & Mason, B. A. (2016). Technology delivered self-monitoring application to promote successful inclusion of an elementary student with autism. *Assistive Technology*, *28*(1), 9‑16. <https://doi.org/10.1080/10400435.2015.1059384>

Rothschild, L. B., Ratto, A. B., Kenworthy, L., Hardy, K. K., Verbalis, A., Pugliese, C., Strang, J. F., Safer‐Lichtenstein, J., Anthony, B. J., Anthony, L. G., Guter, M. M., & Haaga, D. A. F. (2022). Parents matter : Parent acceptance of school‐based executive functions interventions relates to improved child outcomes. *Journal of Clinical Psychology*, *78*(7), 1388‑1406. <https://doi.org/10.1002/jclp.23309>

Rousseau, V., Richard, V., Goupil, G., & Achim, A. (2009). Comportements des élèves de classes ordinaires dans des groupes de jeux intégrés avec des enfants ayant un trouble envahissant du développement. *Revue de psychoéducation*, *38*(1), 1‑14. <https://doi.org/10.7202/1096894ar>

Ruble, L., & McGrew, J. H. (2013). Teacher and Child Predictors of Achieving IEP Goals of Children with Autism. *Journal of Autism and Developmental Disorders*, *43*(12), 2748‑2763. <https://doi.org/10.1007/s10803-013-1884-x>

Sabey, C., Ross, S., & Goodman, J. (2020). Beyond topography : Addressing the functional impact of social skills training for students with autism. *Educational Psychology in Practice*, *36*(2), 133‑148. <https://doi.org/10.1080/02667363.2019.1703650>

Sainato, D. M., Morrison, R. S., Jung, S., Axe, J., & Nixon, P. A. (2015). A Comprehensive Inclusion Program for Kindergarten Children With Autism Spectrum Disorder. *Journal of Early Intervention*, *37*(3), 208‑225. <https://doi.org/10.1177/1053815115613836>

Saleur, C., Tazouti, Y., & Luxembourger, C. (2021). Ralentir la parole pour favoriser la compréhension verbale des enfants porteurs d’autisme ? *Neuropsychiatrie de l’Enfance et de l’Adolescence*, *69*(5), 228‑234. <https://doi.org/10.1016/j.neurenf.2021.06.004>

Sam, A. M., Odom, S. L., Tomaszewski, B., Perkins, Y., & Cox, A. W. (2021). Employing Evidence-Based Practices for Children with Autism in Elementary Schools. *Journal of Autism and Developmental Disorders*, *51*(7), 2308‑2323. <https://doi.org/10.1007/s10803-020-04706-x>

Santarosa, L. M. C., & Conforto, D. (2016). Educational and digital inclusion for subjects with autism spectrum disorders in 1:1 technological configuration. *Computers in Human Behavior*, *60*, 293‑300. <https://doi.org/10.1016/j.chb.2016.02.021>

Scheil, K. A., Bowers-Campbell, J., & Campbell, J. M. (2017). An Initial Investigation of the Kit for Kids Peer Educational Program. *Journal of Developmental and Physical Disabilities*, *29*(4), 643‑662. <https://doi.org/10.1007/s10882-017-9540-6>

Shih, W., Dean, M., Kretzmann, M., Locke, J., Senturk, D., Mandell, D. S., Smith, T., & Kasari, C. (2019). Remaking Recess Intervention for Improving Peer Interactions at School for Children With Autism Spectrum Disorder : Multisite Randomized Trial. *School Psychology Review*, *48*(2), 133‑144. <https://doi.org/10.17105/SPR-2017-0113.V48-2>

Shire, S. Y., Shih, W., Chang, Y.-C., Bracaglia, S., Kodjoe, M., & Kasari, C. (2019). Sustained Community Implementation of JASPER Intervention with Toddlers with Autism. *Journal of Autism and Developmental Disorders*, *49*(5), 1863‑1875. <https://doi.org/10.1007/s10803-018-03875-0>

Shochet, I. M., Saggers, B. R., Carrington, S. B., Orr, J. A., Wurfl, A. M., Kelly, R. L., & Duncan, B. M. (2022). A School-Based Approach to Building Resilience and Mental Health Among Adolescents on the Autism Spectrum : A Longitudinal Mixed Methods Study. *School Mental Health*, *14*(3), 753‑775. <https://doi.org/10.1007/s12310-022-09501-w>

Shogren, K. A., Lang, R., Machalicek, W., Rispoli, M. J., & O’Reilly, M. (2011). Self- Versus Teacher Management of Behavior for Elementary School Students With Asperger Syndrome : Impact on Classroom Behavior. *Journal of Positive Behavior Interventions*, *13*(2), 87‑96. <https://doi.org/10.1177/1098300710384508>

Smith, B. R., Spooner, F., & Wood, C. L. (2013). Using embedded computer-assisted explicit instruction to teach science to students with autism spectrum disorder. *Research in Autism Spectrum Disorders*, *7*(3), 433‑443. <https://doi.org/10.1016/j.rasd.2012.10.010>

Spaniol, M. M., Shalev, L., Kossyvaki, L., & Mevorach, C. (2018). Attention Training in Autism as a Potential Approach to Improving Academic Performance : A School-Based Pilot Study. *Journal of Autism and Developmental Disorders*, *48*(2), 592‑610. <https://doi.org/10.1007/s10803-017-3371-2>

Srivastava, M., De Boer, A. A., & Pijl, S. J. (2015). Know How to Teach Me… Evaluating the Effects of an In-Service Training Program for Regular School Teachers Toward Inclusive Education. *International Journal of School & Educational Psychology*, *3*(4), 219‑230. <https://doi.org/10.1080/21683603.2015.1064841>

Stamou, A., Roussy, A. B., Ockelford, A., & Terzi, L. (2022). Music and dance enhance social interaction and task engagement in autistic young pupils and their peers in mainstream schools. *Support for Learning*, *37*(3), 450‑463. <https://doi.org/10.1111/1467-9604.12420>

Staniland, J. J., & Byrne, M. K. (2013). The Effects of a Multi-Component Higher-Functioning Autism Anti-Stigma Program on Adolescent Boys. *Journal of Autism and Developmental Disorders*, *43*(12), 2816‑2829. <https://doi.org/10.1007/s10803-013-1829-4>

Strogilos, V., & Avramidis, E. (2016). Teaching experiences of students with special educational needs in co‐taught and non‐co‐taught classes. *Journal of Research in Special Educational Needs*, *16*(1), 24‑33. <https://doi.org/10.1111/1471-3802.12052>

Sulek, R., Trembath, D., Paynter, J., & Keen, D. (2019). Social validation of an online tool to support transitions to primary school for children with autism. *Research in Autism Spectrum Disorders*, *66*, 101408. <https://doi.org/10.1016/j.rasd.2019.101408>

Sutton, B. M., Westerveld, M. F., & Webster, A. A. (2022). Classroom Teachers’ Implementation of the Social Stations Intervention to Improve the Verbal Initiations and Responses of Students with Autism. *Journal of Autism and Developmental Disorders*, *52*(3), 1268‑1282. <https://doi.org/10.1007/s10803-021-05042-4>

Symes, W., & Humphrey, N. (2012). Including pupils with autistic spectrum disorders in the classroom : The role of teaching assistants. *European Journal of Special Needs Education*, *27*(4), 517‑532. <https://doi.org/10.1080/08856257.2012.726019>

Taliaferro, A. R., Hammond, L., & Wyant, K. (2015). Preservice Physical Educators’ Self-Efficacy Beliefs Toward Inclusion : The Impact of Coursework and Practicum. *Adapted Physical Activity Quarterly*, *32*(1), 49‑67. <https://doi.org/10.1123/apaq.2013-0112>

Toelken, S., & Miltenberger, R. G. (2012). Increasing Independence Among Children Diagnosed With Autism Using A Brief Embedded Teaching Strategy. *Behavioral Interventions*, *27*(2), 93‑104. <https://doi.org/10.1002/bin.337>

Tupou, J., Waddington, H., & Sigafoos, J. (2022). Teachers’ Perceptions of an Early Intervention Coaching Program. *Advances in Neurodevelopmental Disorders*, *6*(4), 506‑520. <https://doi.org/10.1007/s41252-022-00267-5>

Tupou, J., Waddington, H., Van Der Meer, L., & Sigafoos, J. (2022). Effects of a low-intensity Early Start Denver Model-based intervention delivered in an inclusive preschool setting. *International Journal of Developmental Disabilities*, *68*(2), 107‑121. <https://doi.org/10.1080/20473869.2019.1707434>

Tzanakaki, P., Grindle, C. F., Dungait, S., Hulson-Jones, A., Saville, M., Hughes, J. C., & Hastings, R. P. (2014). Use of a tactile prompt to increase social initiations in children with autism. *Research in Autism Spectrum Disorders*, *8*(6), 726‑736. <https://doi.org/10.1016/j.rasd.2014.03.016>

Vargo, K., & Brown, C. (2020). An evaluation of and preference for variations of the Good Behavior Game with students with autism. *Behavioral Interventions*, *35*(4), 560‑570. <https://doi.org/10.1002/bin.1740>

Vincent, L. B., Asmus, J. M., Lyons, G. L., Born, T., Leamon, M., DenBleyker, E., & McIntire, H. (2022). Evaluating the effectiveness of a reverse inclusion Social Skills intervention for children on the Autism Spectrum. *Journal of Autism and Developmental Disorders*, *53*(7), 2647‑2662. <https://doi.org/10.1007/s10803-022-05513-2>

Vincent, L. B., Openden, D., Gentry, J. A., Long, L. A., & Matthews, N. L. (2018). Promoting Social Learning at Recess for Children with ASD and Related Social Challenges. *Behavior Analysis in Practice*, *11*(1), 19‑33. <https://doi.org/10.1007/s40617-017-0178-8>

Visser, J., & Dubsky, R. (2009). Peer attitudes to SEBD in a secondary mainstream school. *Emotional and Behavioural Difficulties*, *14*(4), 315‑324. <https://doi.org/10.1080/13632750903303153>

Vivanti, G., Dissanayake, C., Duncan, E., Feary, J., Capes, K., Upson, S., Bent, C. A., Rogers, S. J., Hudry, K., Jones, C., Bajwa, H., Marshall, A., Maya, J., Pye, K., Reynolds, J., Rodset, D., & Toscano, G. (2019). Outcomes of children receiving Group-Early Start Denver Model in an inclusive versus autism-specific setting : A pilot randomized controlled trial. *Autism*, *23*(5), 1165‑1175. <https://doi.org/10.1177/1362361318801341>

Waddington, E. M., & Reed, P. (2009). The impact of using the “Preschool Inventory of Repertoires for Kindergarten” (PIRK®) on school outcomes of children with Autistic Spectrum Disorders. *Research in Autism Spectrum Disorders*, *3*(3), 809‑827. <https://doi.org/10.1016/j.rasd.2009.03.002>

Wang, H.-I., Wright, B. D., Bursnall, M., Cooper, C., Kingsley, E., Le Couteur, A., Teare, D., Biggs, K., McKendrick, K., De La Cuesta, G. G., Chater, T., Barr, A., Solaiman, K., Packham, A., Marshall, D., Varley, D., Nekooi, R., Gilbody, S., & Parrott, S. (2022). Cost-utility analysis of LEGO based therapy for school children and young people with autism spectrum disorder : Results from a randomised controlled trial. *BMJ Open*, *12*(1), e056347. <https://doi.org/10.1136/bmjopen-2021-056347>

Ward, P., & Ayvazo, S. (2006). Classwide Peer Tutoring in Physical Education : Assessing Its Effects With Kindergartners With Autism. *Adapted Physical Activity Quarterly*, *23*(3), 233‑244.

Watkins, L., O’Reilly, M., Kuhn, M., & Ledbetter‐Cho, K. (2019). An interest‐based intervention package to increase peer social interaction in young children with autism spectrum disorder. *Journal of Applied Behavior Analysis*, *52*(1), 132‑149. <https://doi.org/10.1002/jaba.514>

Williams, N. J., Frederick, L., Ching, A., Mandell, D., Kang-Yi, C., & Locke, J. (2021). Embedding school cultures and climates that promote evidence-based practice implementation for youth with autism : A qualitative study. *Autism*, *25*(4), 982‑994. <https://doi.org/10.1177/1362361320974509>

Wilson, W. J., Harper-Hill, K., Armstrong, R., Downing, C., Perrykkad, K., Rafter, M., & Ashburner, J. (2021). A preliminary investigation of sound-field amplification as an inclusive classroom adjustment for children with and without Autism Spectrum Disorder. *Journal of Communication Disorders*, *93*, 106-142. <https://doi.org/10.1016/j.jcomdis.2021.106142>

Wolfberg, P., DeWitt, M., Young, G. S., & Nguyen, T. (2015). Integrated Play Groups : Promoting Symbolic Play and Social Engagement with Typical Peers in Children with ASD Across Settings. *Journal of Autism and Developmental Disorders*, *45*(3), 830‑845. <https://doi.org/10.1007/s10803-014-2245-0>

Wong, C. S. (2013). A play and joint attention intervention for teachers of young children with autism : A randomized controlled pilot study. *Autism*, *17*(3), 340‑357. <https://doi.org/10.1177/1362361312474723>

Woodcock, A., Woolner, A., & Benedyk, R. (2009). Applying the Hexagon-Spindle Model to the design of school environments for children with Autistic spectrum disorders. *Work*, *32*(3), 249‑259. <https://doi.org/10.3233/WOR-2009-0823>

Wright, B., Marshall, D., Collingridge Moore, D., Ainsworth, H., Hackney, L., Adamson, J., Ali, S., Allgar, V., Cook, L., Dyson, L., Littlewood, E., Hargate, R., McLaren, A., McMillan, D., Trepel, D., Whitehead, J., & Williams, C. (2014). Autism Spectrum Social Stories In Schools Trial (ASSSIST) : Study protocol for a feasibility randomised controlled trial analysing clinical and cost-effectiveness of Social Stories in mainstream schools. *BMJ Open*, *4*(7), e005952‑e005952. <https://doi.org/10.1136/bmjopen-2014-005952>

Wu, Y.-C., Chen, P.-Y., Tsai, S.-P., Tsai, S.-F., Chou, Y.-C., & Chiu, C.-Y. (2019). The effects of the class-wide function-related intervention teams on behaviors of an elementary student with autism spectrum disorder in an inclusive classroom in Taiwan. *International Journal of Developmental Disabilities*, *65*(5), 368‑377. <https://doi.org/10.1080/20473869.2019.1647031>

Xu, S., Wang, J., Lee, G. T., & Luke, N. (2017). Using Self-Monitoring With Guided Goal Setting to Increase Academic Engagement for a Student With Autism in an Inclusive Classroom in China. *The Journal of Special Education*, *51*(2), 106‑114. <https://doi.org/10.1177/0022466916679980>

Young, K. R., Radley, K. C., Jenson, W. R., West, R. P., & Clare, S. K. (2016). Peer-facilitated discrete trial training for children with autism spectrum disorder. *School Psychology Quarterly*, *31*(4), 507‑521. <https://doi.org/10.1037/spq0000161>

Yuan, S. N. V., & Ip, H. H. S. (2018). Using virtual reality to train emotional and social skills in children with autism spectrum disorder. *London Journal of Primary Care*, *10*(4), 110‑112. <https://doi.org/10.1080/17571472.2018.1483000>
